# Integrated investigation and experimental validation of PPARG as an oncogenic driver: implications for prognostic assessment and therapeutic targeting in hepatocellular carcinoma

**DOI:** 10.3389/fphar.2023.1298341

**Published:** 2023-11-15

**Authors:** Yunsheng Ran, Chujiao Hu, Junzhao Wan, Qian Kang, Ruixian Zhou, Ping Liu, Dan Ma, Jianta Wang, Lei Tang

**Affiliations:** ^1^ School of Pharmacy, Guizhou Medical University, Guiyang, China; ^2^ Guizhou Provincial Engineering Technology Research Center for Chemical Drug R&D, Guizhou Medical University, Guiyang, China; ^3^ State Key Laboratory of Functions and Applications of Medicinal Plants, Guizhou Medical University, Guiyang, China; ^4^ Department of Hematology, Affiliated Hospital of Guizhou Medical University, Guiyang, China; ^5^ Department of Acupuncture and Moxibustion, Affiliated Hospital of Guizhou Medical University, Guiyang, China; ^6^ Key Laboratory of Endemic and Ethnic Diseases, Ministry of Education and Key Laboratory of Medical Molecular Biology of Guizhou Province, Guizhou Medical University, Guiyang, Guizhou, China

**Keywords:** PPARG, prognosis, tumor microenvironment, drug sensitivity, immunological function, biomarkers

## Abstract

Peroxisome proliferator-activated receptor gamma (PPARG), a key transcription factor involved in lipid metabolism and glucose homeostasis, has been implicated in various types of cancer. However, its precise role in cancer remains unclear. In this study, we conducted a comprehensive pan-cancer analysis of PPARG expression using various types of cancer obtained from public databases. We observed significant heterogeneity in PPARG expression across different types of cancer. The association between PPARG expression and patient prognosis was investigated using Cox proportional hazards regression models and survival analysis. Clinical features and protein expression levels in the cohort showed that PPARG expression was strongly associated, suggesting its potential as a therapeutic target. We also evaluated the prognostic potential of PPARG by analyzing immune infiltration and genomic stability. We experimentally validated the potential of PPARG as a therapeutic target by analyzing drug sensitivity profiles, molecular docking simulations, and *in vitro* cell proliferation assays associated with PPARG expression. We identified common expression patterns of PPARG with other genes involved in key carcinogenic pathways. This provides deeper insights into the molecular mechanisms underlying its carcinogenic role. Additionally, functional enrichment analysis revealed significant enrichment of genes related to drug metabolism, cell proliferation, and immune response pathways associated with PPARG. Our findings highlight the importance of PPARG in the broader biology of cancer and suggest its potential as a diagnostic and therapeutic target for specific types of cancer. The results of our study provide strong support for the potential role of PPARG as a promising prognostic biomarker and immunotherapeutic target across various types of cancer.

## 1 Introduction

Cancer has emerged as the leading cause of global mortality and presents a formidable barrier to enhancing both the quality and quantity of human life in the 21st century (Bray et al., 2018). The prevalence and intricate progression of tumors have presented significant challenges in the fields of cancer prevention, diagnosis, and treatment. Consequently, comprehensive research and exploration of biomarkers related to cancer, along with a deep understanding of their roles in cancer, elucidation of their functional significance, and evaluation of their potential as prognostic and predictive indicators, offer significant potential for advancing our understanding of the disease and enhancing therapeutic strategies (Wild, 2019).

Peroxisome proliferator-activated receptors (PPARs) were initially discovered in 1990 by British scientists Issemann et al. [3] as ligand-activated intranuclear transcription factors. There are three known subtypes of PPAR: PPAR-alpha (α), PPAR-delta (δ), and PPAR-gamma (γ). Among these subtypes, PPARγ, encoded by the *PPARG* gene, acts as a regulator of adipocyte differentiation. Recently, the association between PPAR proteins and cancer has garnered significant attention. PPARγ, a member of the nuclear receptor superfamily, plays a crucial role in controlling various biological processes (BPs), such as cell division, differentiation, and inflammation (Shu et al., 2016; Kesanakurti et al., 2017). Furthermore, PPARG exhibits widespread expression, particularly in the liver, colon, heart, several types of epithelial cells, and skeletal muscle (Verreth et al., 2004; Lewis et al., 2008; Zhang et al., 2012; Yang et al., 2016). As a transcription factor, PPARG is involved in the regulation of lipid anabolism and glycolysis to meet the energy demands of cancer cells. The metabolic reprogramming of glucose, lipids, and amino acid metabolism during tumorigenesis provides essential energy and substrates for sustained tumor cell proliferation and survival (Menezes and Diederich, 2021; Villarroel-Vicente et al., 2021). The activation of PPARG has been associated with beneficial effects on various types of cancer. It has been established that in cancer cells, the activation of PPARG leads to cell cycle arrest, suppression of cell proliferation, and increased apoptosis. (Mal et al., 2021). Additionally, the activation of PPARG has demonstrated anti-inflammatory properties by suppressing the production of pro-inflammatory cytokines and modulating the tumor microenvironment (TME) (Shao et al., 2020). Intriguingly, synthetic compounds known as PPARγ agonists, which activate PPARG, have shown promise as potential therapeutic agents in the treatment of cancer (Ryu et al., 2018; Ballav et al., 2022). Both preclinical and clinical studies have reported the anticancer effects of PPARγ agonists, including inhibition of tumor growth and reduced angiogenesis (Hughes et al., 2014; Joshi et al., 2014). However, the role of PPARG in tumor initiation remains highly intricate and is influenced by environmental factors (Vallee et al., 2018). The significance of PPARG in cancer development and the potential for therapeutic targeting of PPARG remain elusive.

This study aims to comprehensively investigate PPARG expression in human cancers and its impact on prognosis and clinical significance. Additionally, we sought to elucidate the association between PPARG and tumor formation, grading, and metastasis. We examined the expression features of PPARG in cancer, including its effects on genomic stability, the immune microenvironment, and potential molecular mechanisms. Furthermore, cell functional experiments were conducted to confirm the feasibility of targeting PPARG as a potential anticancer therapy. Understanding the intricate interplay between PPARG and tumors holds tremendous potential for the development of novel therapeutic strategies that target this pathway. By exploring the molecular mechanisms underlying the involvement of PPARG in tumorigenesis, we can uncover new avenues for the treatment of cancer and potentially identify biomarkers for patient stratification and personalized therapeutic approaches.

## 2 Materials and methods

### 2.1 Data collection and processing

The original raw data were obtained from The Cancer Genome Atlas (TCGA) (accessible at https://cancergenome.nih.gov/) and the Gene Expression Omnibus (GEO) (accessible at https://www.ncbi.nlm.nih.gov/geo/) databases. The obtained data were processed and cleaned separately using the following portals.

### 2.2 PPARG differential expression analysis in 33 human cancers

To assess the PPARG differential expression in various human cancers, the Tumor IMmune Estimation Resource (TIMER) database (accessible at https://cistrome.shinyapps.io/timer/) and the Gene Expression Profiling Interactive Analysis (GEPIA) database (accessible at http://gepia2.cancer-pku.cn/analysis/)were used. These databases allowed the measurement of PPARG expression in tumor tissues compared to normal tissues across different types of cancer. The differences in mRNA expression levels were analyzed, and the distributions of gene expression levels were visualized using box plots.

### 2.3 Survival analysis and prognosis in human cancers

The Kaplan-Meier method of survival analysis was used to analyze overall survival (OS), disease-specific survival (DSS), progression-free interval (PFI), and disease-free interval (DFI). The R software package Survival (version 3.2–7) was used for this analysis. Cox proportional hazards regression models, implemented through the Coxph function, were used to analyze the association between gene expression and prognosis in each type of cancer. The log-rank test was used to perform statistical tests to obtain prognostic significance.

### 2.4 Genetic alteration analysis

The online platform cBioportal (accessible at https://www.cbioportal.org/) was used to analyze the genetic variation features of PPARG. This analysis involved investigating the query mutation frequency, types of mutation, and post-translational modifications (PTM).

### 2.5 Estimate of the TME, tumor mutational burden, microsatellite instability, and PPARG expression levels and immunological checkpoint genes in human cancers

Sanger Box, an online tool for TCGA data processing, was used to investigate the association between PPARG expression and immunological checkpoint (ICP) genes, biomarkers, and predicted scores in the TME. The unified and standardized pan-cancer dataset was obtained from the University of California Santa Cruz (UCSC; accessible at https://xenabrowser.net/) database. The dataset used was TCGA TARGET GTEx (PANCAN, N = 19,131, G = 60,499). From this dataset, the gene expression data of PPARG in each sample was extracted. The tumor mutational burden (TMB) of each tumor was calculated using the tmb function from the R package maftools (version 2.8.05). Additionally, the microsatellite instability (MSI) score of each tumor was determined by consulting previous studies.

### 2.6 Examination of the interaction between PPARG expression and clinical features

RNA-sequencing and clinical data for 31 types of cancer are collected from the UALCAN database (accessible at http://ualcan.path.uab.edu), which provides a reliable platform for examining gene expression in both tumors and healthy tissues. This database was utilized to explore the correlation between specific gene expression levels and clinicopathological features in human tumors.

### 2.7 Investigating PPARG co-expression networks using a database

The database LinkedOmics (accessible at http://www.linkedomics.org/) provides public access to multi-omics data from all 32 TCGA types of cancer and 10 Clinical Proteomics Tumor Analysis Consortium (CPTAC) cancer cohorts. Pearson’s correlation coefficient was used to identify the PPARG co-expressed genes, and the results were visualized through a heat map, volcano plot, and table. Furthermore, gene set enrichment analysis (GSEA) was conducted to explore the association between the Kyoto Encyclopedia of Genes and Genomes (KEGG) pathway and the Gene Ontology (GO) BPs associated with PPARG and its co-expressed genes.

### 2.8 Sample collection

A total of 20 tissue samples, including 10 pancreatic cancer samples and 10 liver cancer samples, were collected from patients at the Pathology Department of Guizhou Medical University Affiliated Hospital. All tissue wax blocks underwent histological diagnosis following the guidelines of the World Health Organization (WHO). The study was approved by the ethics committee of Guizhou Medical University (Approval No. 538, 2023) and adhered to the principles outlined in the Helsinki Declaration. According to Chinese law, the materials used in this study were collected by the Pathology Department of Guizhou Medical University Affiliated Hospital for the purposes of diagnosis and treatment, and are used in an anonymous form for this research. Therefore, written consent from patients is not required.

### 2.9 Immunohistochemical staining for PPARG expression in tumor tissue

Immunohistochemical staining was performed using an immunohistochemical kit (Zhongshan Jinqiao, Beijing, China). Paraffin sections were dewaxed at 65°C and subsequently dewaxed using gradient concentrations of xylene and ethanol. Antigen repair was conducted by high-pressure heating with a citric acid solution (Servicebio, Wuhan, China) at pH 9.0 for 5 min. Following the removal of endogenous peroxidase using the peroxidase blocker from the immunohistochemistry kit and blocking non-specific antigen with 10% goat serum (Booster, Wuhan, China), the primary antibody (1:200) was added and incubated overnight at 4°C. The secondary antibody reaction enhancement solution in the kit was incubated for 30 min at 37°C, followed by a 30-min incubation with goat or mouse anti-rabbit immunoglobulin G (IgG) at 37°C. The slides were developed using diaminobenzidine (DAB; Servicebio, Wuhan, China) reagent, counterstained with hematoxylin, fractionated using 1% hydrochloric acid in alcohol for 10 s, and then blued with a saturated lithium carbonate solution. Finally, the slides were sealed with neutral gum and photographed using an ortho-optical microscope (Nikon). Additionally, we obtained immunohistochemistry (IHC) images depicting the protein expression levels of PPARG in LIHC and PAAD from the tissue and pathology modules of the Human Protein Atlas (accessible at https://www.proteinatlas.org/).

### 2.10 Cell culture

Hepatocellular carcinoma cells (LM3, Huh7, HLF, Hep G2) and normal liver control cells (LO2 and L68) were generously provided by Dr. Chu Jiao Hu from the Guizhou Provincial Engineering Technology Research Center for Chemical Drug R&D, Guizhou Medical University. The cells were cultured under the following conditions: Dulbecco’s modified Eagle medium (Gibco), supplemented with 10% fetal bovine serum (FBS; Cell Box, China). Briefly, the cells were cultured in T25 cell culture flasks at 37°C in a constant temperature incubator with 5% CO_2_. When the cell density reached 70%–90%, trypsin digestion with 0.25% ethylenediaminetetraacetic acid (EDTA; Servicebio, Wuhan, China) was used for a 3-min incubation to detach the cells for subsequent experiments.

### 2.11 Gene expression and drug sensitivity profiling analysis

The analysis involved utilizing the specialist R software OncoPredict, which combines gene expression profiles with drug sensitivity profiles, to assess the drug sensitivity data of relevant cancer cohorts from the TCGA database. Additionally, expression matrices from the GSE14520 and TCGA-LIHC datasets were obtained from the GEO database for the PPARG expression and drug sensitivity analyses.

### 2.12 Molecular docking

Molecular docking was performed using the AutoDock software, employing specific branches (AutoDock v4.2.6, Autodock Vina, and Autodocktools v1.5.6) for the docking of protein receptors with small-molecule ligands. The three-dimensional (3D) structures of four small molecule compounds (BDP9066, Axitinib, Nilotinib, and Dabrafenib) in SDF format were retrieved from PubChem (accessible at https://pubchem.ncbi.nlm.nih.gov/) and analyzed using SYBYL-X 2.0 for the receptor and small molecule ligand processing, resulting in energy-minimized optimal conformations. Based on previous studies, the three-dimensional spatial locations of the active pocket in the PPARγ protein receptor (PDB: 6K0T) responsible for inhibition of tumor proliferation (Yamamoto et al., 2019), at which were determined using Autodocktools v1.5.6 software with centers of −1.063, 13.081, and 22.373 with sizes of 20 Å, 20 Å, and 20 Å, respectively. The four small-molecule ligands underwent docking analysis using Autodock Vina v1.2.3 software, following the principles of semi-flexible docking. The optimal conformation from the docking results was selected for further analysis and processing using PYMOL v2.1.1 software to generate images.

### 2.13 Western blot analysis

Western blot analysis was conducted following a previously described protocol (Ma et al., 2023). Protein lysates from whole cells were obtained by using radioimmunoprecipitation assay (RIPA) lysis buffer supplemented with protease inhibitors, and equivalent amounts of protein were loaded onto a 10% sodium dodecyl sulfate-polyacrylamide gel electrophoresis gel. The primary antibodies used in the analysis included rabbit anti-PPARγ (Immunoway, SuZhou, China) and rabbit anti-β-actin (Santa Cruz). The membranes were incubated overnight at 4°C with the primary antibodies, followed by incubation with horseradish peroxidase-conjugated anti-rabbit IgG as the secondary antibody. Chemiluminescence detection was used to visualize the protein bands, and images were captured using a BIO-RAD imager. The greyscale values of the bands were quantified using ImageJ software.

### 2.14 CCK-8 assay for proliferation and viability of hepatocellular carcinoma cells

After trypsinization and digestion with 0.25% EDTA, the cells were counted and evenly distributed into 96-well plates at a density of 5,000 cells per well. Following a 24-h cell attachment period, the culture medium was replaced with a basal medium containing the appropriate drug concentration. The cells were then incubated under standard cell culture conditions for 24 h. Subsequently, 10 µL of CCK-8 reagent (Abisin, Shanghai, China) was added to each well and incubated for 2 h. The drugs used in the experiment were Axitinib (Aladdin, Shanghai, China), Nilotinib (SelleckChem, Houston, United States), Dabrafenib (Aladdin, Shanghai, China), and BDP9066 (GlpBio, Shanghai, China). The absorbance of each drug concentration was measured at a wavelength of 450 nm using a Microplate reader (Thermo Fisher Scientific Inc., Shanghai, China).

### 2.15 Statistics analysis

Various investigations, including TBM, MSI, immunological checkpoints, molecular biology, and bioinformatics, were conducted to validate the association between PPAR expression and the research objectives, such as survival prognosis and clinical features. The Pearson test was used to assess the association between PPARG expression and these variables. To compare the expression differences across groups, paired *t-*tests or unpaired *t*-tests were used depending on whether the samples were paired. Statistical significance was defined as *p* < 0.05. R tools were used for data visualization and creating plots, while GraphPad Prism v8.3.0 (San Diego, United States of America) was used for all analyses. For statistical significance analysis involving more than two groups, one-way analysis of variance (ANOVA) was utilized. Two-tailed tests were used to calculate each *p*-value, and a result of *p* < 0.05 was considered statistically significant.

## 3 Results

### 3.1 PPARG expression analysis in human cancers

**FIGURE 1:**
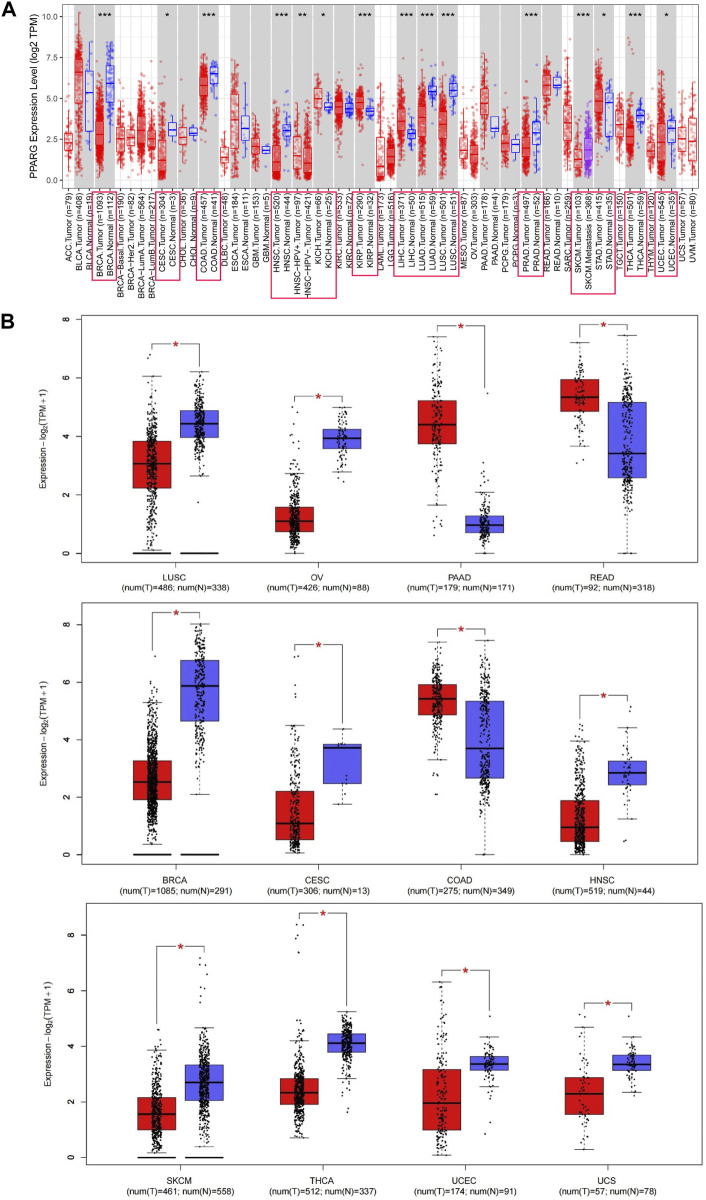
Differential gene expression of PPARG in different human tumor types or specific cancer subtypes. **(A)** Analysis of PPARG expression status in 33 subtypes of cancer by TIMER2 database. **(B)** Differential expression of PPARG in various cancers was analysis through the GEPIA2 database in combination with the TCGA dataset and the GTEx dataset. Log2 (TPM+1) was used as a logarithmic scale. (*p < 0.05, **p < 0.01, and ***p < 0.001).

The PPARG mRNA expression levels in tissue samples with cancer and paraneoplasm were evaluated using the TIMER 2.0 and TCGA databases. In various types of cancer, such as breast cancer (BRCA), cervical cancer (CESC), colon adenocarcinoma (COAD), head and neck squamous cell carcinoma (HNSC), lung adenocarcinoma (LUAD), lung squamous cell carcinoma (LUSC), skin cutaneous melanoma (SKCM), prostate adenocarcinoma (PRAD), thyroid cancer (THCA), and endometrioid cancer (UCEC), PPARG mRNA expression levels were significantly lower in cancerous tissues compared to paraneoplastic tissues. Conversely, in kidney chromophobe (KICH), kidney renal papillary cell carcinoma (KIRP), stomach cancer (STAD), and liver hepatocellular carcinoma (LIHC), there was a significantly higher PPARG expression level in cancerous tissues compared to paraneoplastic tissues (Figure 1A). However, due to the limited number of normal samples in the TCGA database and potential variations across data center platforms, we combined the normal tissue data from the GTEx database with the tumor tissue data in the GEPIA2 online database. This approach enabled us to analyze the differences in PPARG mRNA expression across 27 types of cancer. As shown in Figure 1B, the results indicated lower PPARG expression levels in nine types of cancer, including LUSC, ovarian serous cystadenocarcinoma (OV), BRCA, CESC, HNSC, SKCM, THCA, UCEC, and uterine carcinosarcoma (UCS). Conversely, PPARG exhibited high expression in pancreatic cancer (PAAD), rectum adenocarcinoma (READ), and COAD. These findings indicate significant heterogeneity in mRNA PPARG expression levels among different human cancers.

### 3.2 Risk proportion of PPARG and prognostic survival analysis in cancer

To investigate the association between PPARG expression level and risk, correlation analysis was conducted between PPARG expression level and survival, including OS, DSS, DFI, and PFI. The Cox proportional hazards model analysis was employed to assess the association between gene expression and prognosis in each type of cancer. The OS analysis (Supplementary Figure S1A) revealed that high PPARG expression was associated with poor prognosis in lower-grade glioma and glioblastoma (GBMLGG), lower-grade glioma (LGG), glioblastoma (GBM), LIHC, THCA, PAAD, and ALL-R. Conversely, in five types of cancer, such as pan-kidney cohort (KIPAN), kidney renal clear cell carcinoma (KIRC), bladder urothelial carcinoma (BLCA), READ, and uveal melanoma (UVM), low PPARG expression was associated with a poor prognosis.

The DSS analysis results showed that high PPARG expression was associated with poor prognosis in six types of cancer, including GBMLGG, LGG, CESC, GBM, THCA, and PAAD. Conversely, in KIPAN, KIRC, BLCA, and UVM, low PPARG expression was associated with poor prognosis (Supplementary Figure S1B). In terms of PFI, forest plots (Supplementary Figure S1C) indicated that high PPARG expression was associated with poor prognosis in GBMLGG, LGG, GBM, and PAAD. However, there was no significant correlation observed between PPARG expression and PFI in KIPAN, KIRC, BLCA, or UVM. DFI data analysis revealed a strong association between high PPARG expression and PAAD, unlike in other human cancers where it was not statistically significant (Supplementary Figure S1D).

**FIGURE 2:**
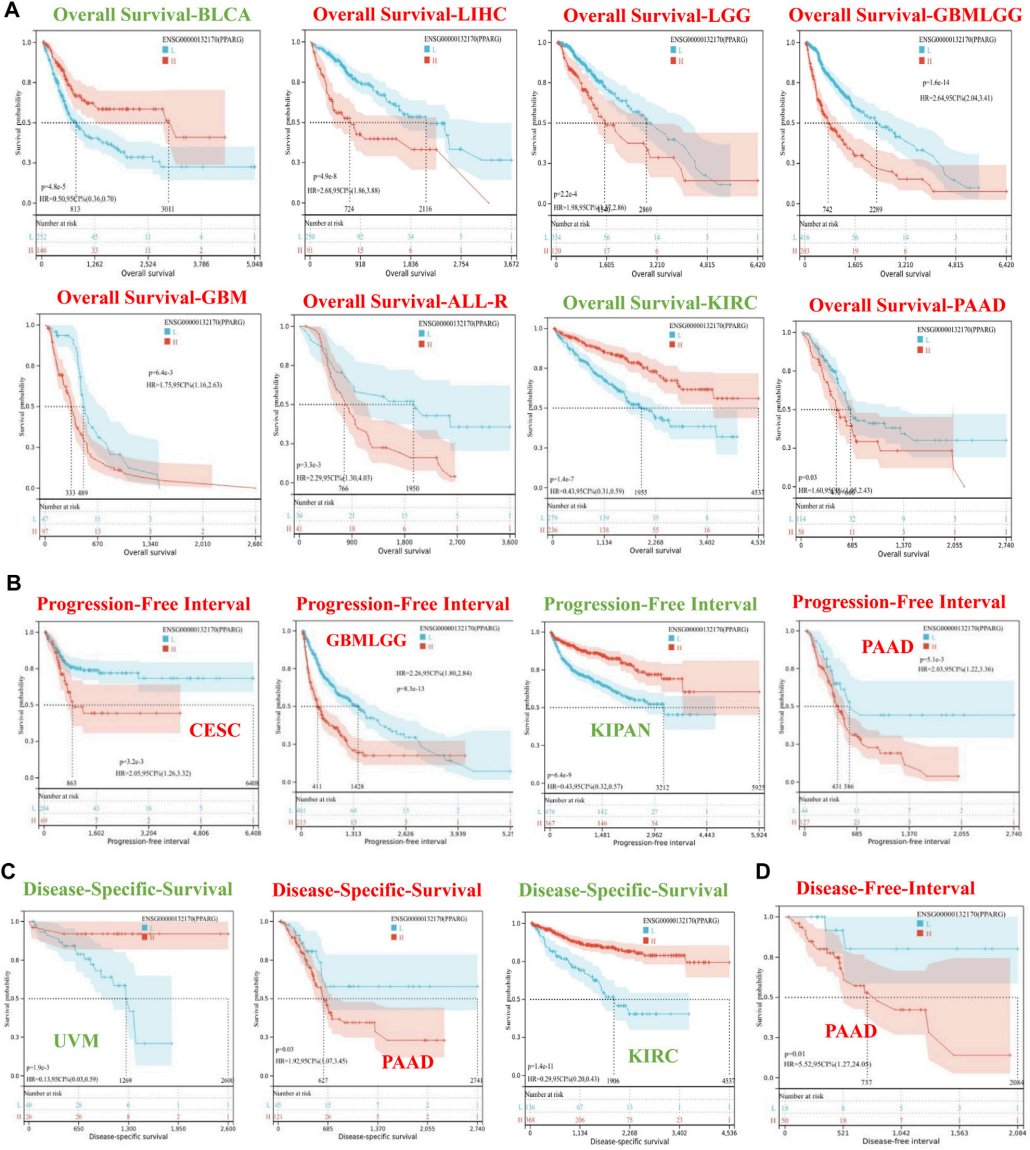
Survival curve of human cancers with high and low PPARG expression analyzed by the Kaplan-Meier method. **(A)** High and low PPARG expression was associated with poorer OS in the pan-cancer analysis. **(B)** Kaplan-Meier analysis of the association between PPARG expression and PFI. **(C)** Kaplan-Meier analysis of the association between PPARG expression and DSS. **(D)** Kaplan-Meier analysis of the association between PPARG expression and DFI.

To determine the appropriate cutoff value for PPARG, we employed the R package maxstat and successfully achieved it. Consequently, the patients were categorized into two groups based on their PPARG expression levels: high and low expression groups. The survfit function in the R software package Survival was used to analyze the prognostic differences between the two groups. The significance of the prognostic difference in OS, PFI, DSS, and DFI among the samples in different groups was evaluated using the log-rank test method (Figure 2). Based on the Kaplan-Meier (KM) survival analysis, patients with LIHC, LGG, GBMLGG, GBM, ALL-R, and PAAD who had high PPARG expression levels had shorter OS compared to those with BLCA and KIRC, who had higher PPARG expression levels and longer OS (Figure 2A). Furthermore, the study revealed that PPARG expression level significantly influenced the survival index of PFI in patients with CESC, GBMLGG, and PAAD (Figure 2B). Additionally, the data indicated that a high PPARG expression level in PAAD was significantly associated with lower DSS and PFI, while high PPARG expression in UVM and KIRC was significantly associated with higher DSS (Figures 2C, D).

### 3.3 Examination of the association between PPARG expression and clinical features

Our findings highlight the variability of PPARG expression across different types of cancer and its impact on patient survival and prognosis. By investigating the correlation between clinical features and gene expression patterns, we can identify molecular biomarkers associated with cancer development and treatment response (Mun et al., 2018). To further explore the association between PPARG expression and clinical features, we used the UALCAN database for RNA-level analysis (Chandrashekar et al., 2022). We observed significant differences in PPARG expression in PAAD and LIHC tumors concerning lymph node metastasis status, histological subtype, molecular subtype, tumor stage, and *TP53* mutation status (Supplementary Figures S2, S3). However, PPARG expression in CESC and LGG is significantly different only in certain clinical features, such as the race of the patient in CESC and histological subtypes and *TP53* mutation status in LGG. PPARG expression in GBM did not exhibit significant differences with any of the clinical features analyzed (Supplementary Figures S4–S6). Additionally, the UALCAN database does not include datasets for relapsed acute lymphoid leukemia, LGG, or glioblastoma. Therefore, analysis and exploration of the association between PPARG expression and clinical features have not been conducted.

### 3.4 PPARG protein expression levels in LIHC and PAAD

**FIGURE 3:**
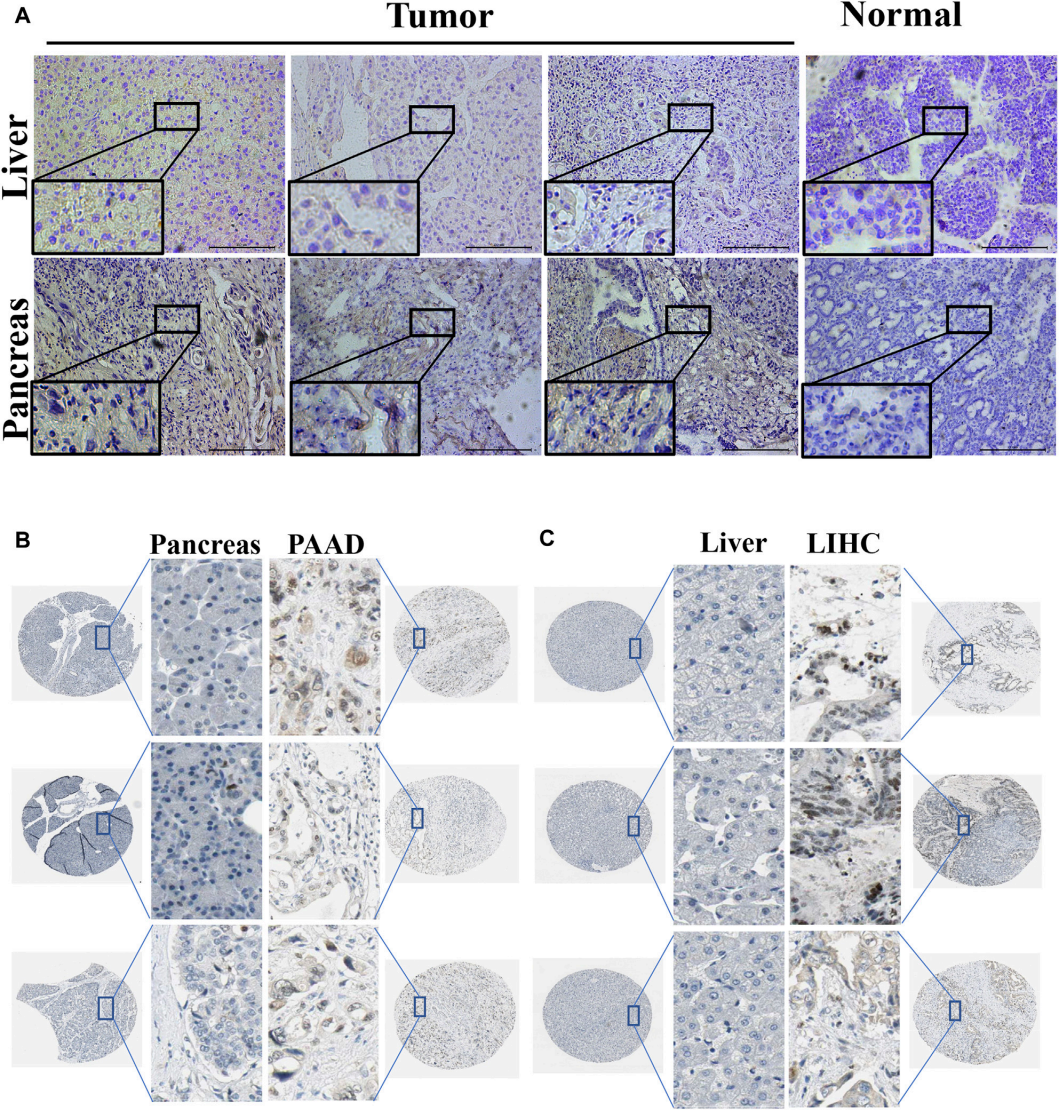
Expression of PPARG in tumor and paraneoplastic tissues. **(A)** Detection of PPARG expression levels in hepatocellular carcinoma and pancreatic cancer by immunohistochemical staining. Scale, 200 um. Images are representatives of three independent experiments with similar results. **(B,C)** Immunohistochemical staining images of PPARG in LIHC and PAAD as well as their normal tissues were collected in the HPA database.

Using immunohistochemistry analysis, we evaluated the protein expression levels of PPARγ, encoded by the *PPARG* gene, in the pathological tissues of patients with PAAD and LIHC. (Figure 3A). The findings demonstrated significantly higher PPARγ protein expression levels in these malignancies compared to the corresponding paracancerous tissues, consistent with the RNA-level expression pattern. To further investigate the variations in PPARγ protein expression levels in LIHC and PAAD, we used the Human Protein Atlas (HPA) online database (Figures 3B, C). The results from the protein-level analysis aligned with the earlier RNA-level study, revealing significantly elevated PPARγ protein expression levels in PAAD and LIHC compared to their respective paracancerous tissues. Finally, these findings indicate that PPARγ is highly expressed in LIHC and PAAD, and this elevated expression is positively associated with adverse disease outcomes and clinical features. Consequently, PPARγ holds promise as a potential target for evaluating cancer prognosis.

### 3.5 PPARG gene alterations analysis in cancer

**FIGURE 4:**
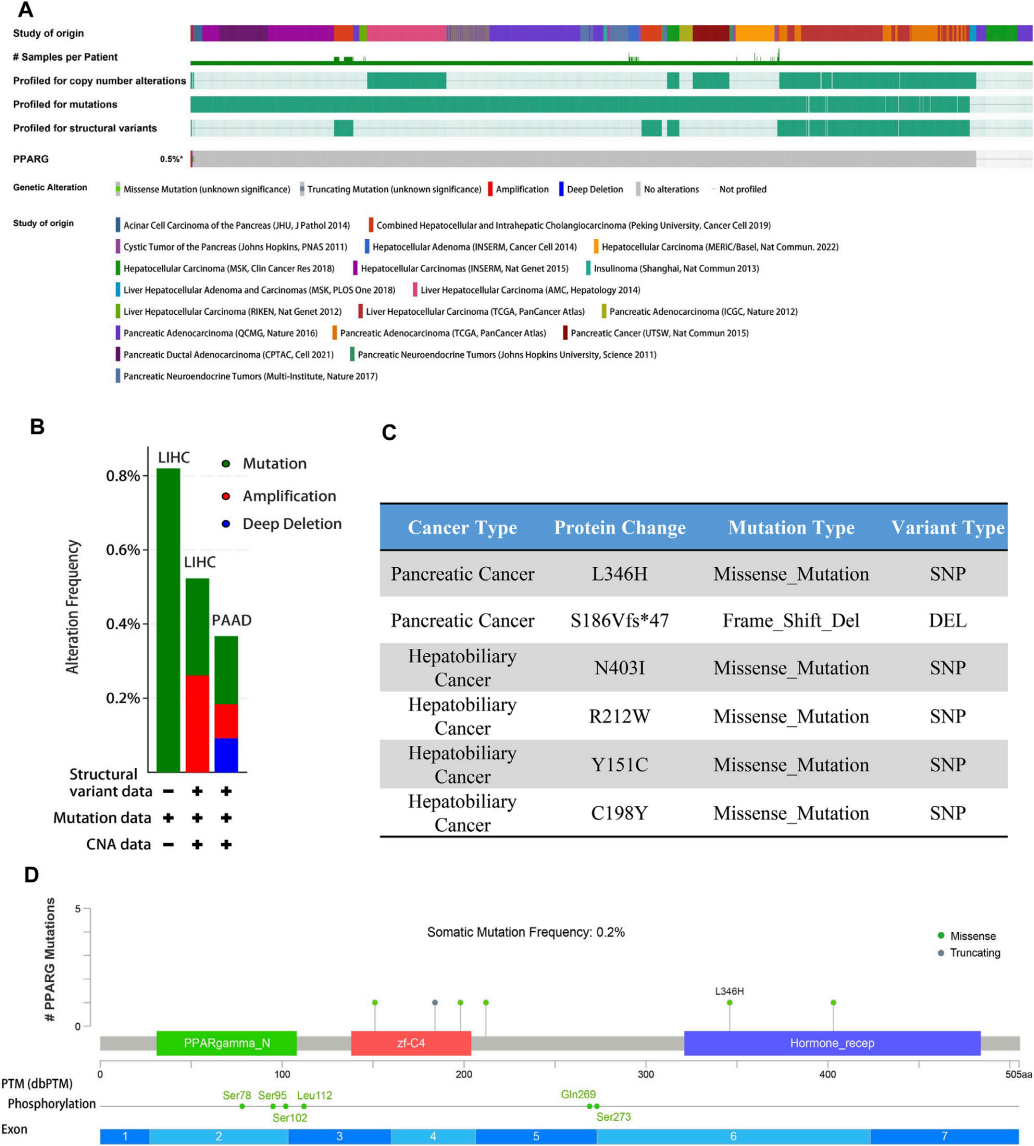
PPARG gene alterations in human cancers analyzed by the cBioPortal database. **(A)** Types of PPARG genetic alterations in different pan-cancer datasets. **(B,C)** Missense mutation, phosphorylation, and acetylation modification of PPARG in the pan-cancer analysis. **(D)** Details of PPARG gene alteration types in the cancer cohort.

Gene alterations play a critical role in the initiation and progression of various types of cancer. Disruptions in the genome can disturb normal cellular processes, leading to uncontrolled growth and tumor formation, ultimately affecting patient prognosis (Baer et al., 2019; Cagan et al., 2022). Understanding the intricate association between gene mutations and cancer is crucial for unraveling the underlying mechanisms driving tumor occurrence, progression, and response to treatment. To explore the association between PPARG expression and frequent somatic mutations, we analyzed 17 datasets of hepatocellular carcinoma and pancreatic cancer from the cBioprotal database, as well as two datasets of pan-cancer. The findings revealed an overall mutation rate of 0.5% (Figure 4A), including missense mutations, deletion mutations, amplifications, and profound deletions. Amplifications were predominantly observed in LIHC, while profound deletions were more prevalent in PAAD (Figure 4B). Despite the high PPARG expression in human cancers, the total mutation rate at the oncogene level was relatively low (0.5%). This suggests that PPARG expression level as a transcription factor may not directly influence chromatin or DNA structural homeostasis. Furthermore, the post-transcriptional modification analysis revealed multiple amino acid phosphorylation sites of PPARG, including amino acid sites Ser78, Ser95, Ser102, Leu112, Gln269, and Ser273 (Figures 4C, D). This suggests a potential role for multisite phosphorylation of PPARG in tumorigenesis. Collectively, these findings indicate the presence of PPARG genetic alterations and differential expression in cancer tissues, particularly in LIHC and PAAD. These observations highlight the potential significance of PPARG in cancer development and progression.

### 3.6 Correlation of PPARG expression levels with genomic stability in LIHC and PAAD

**FIGURE 5:**
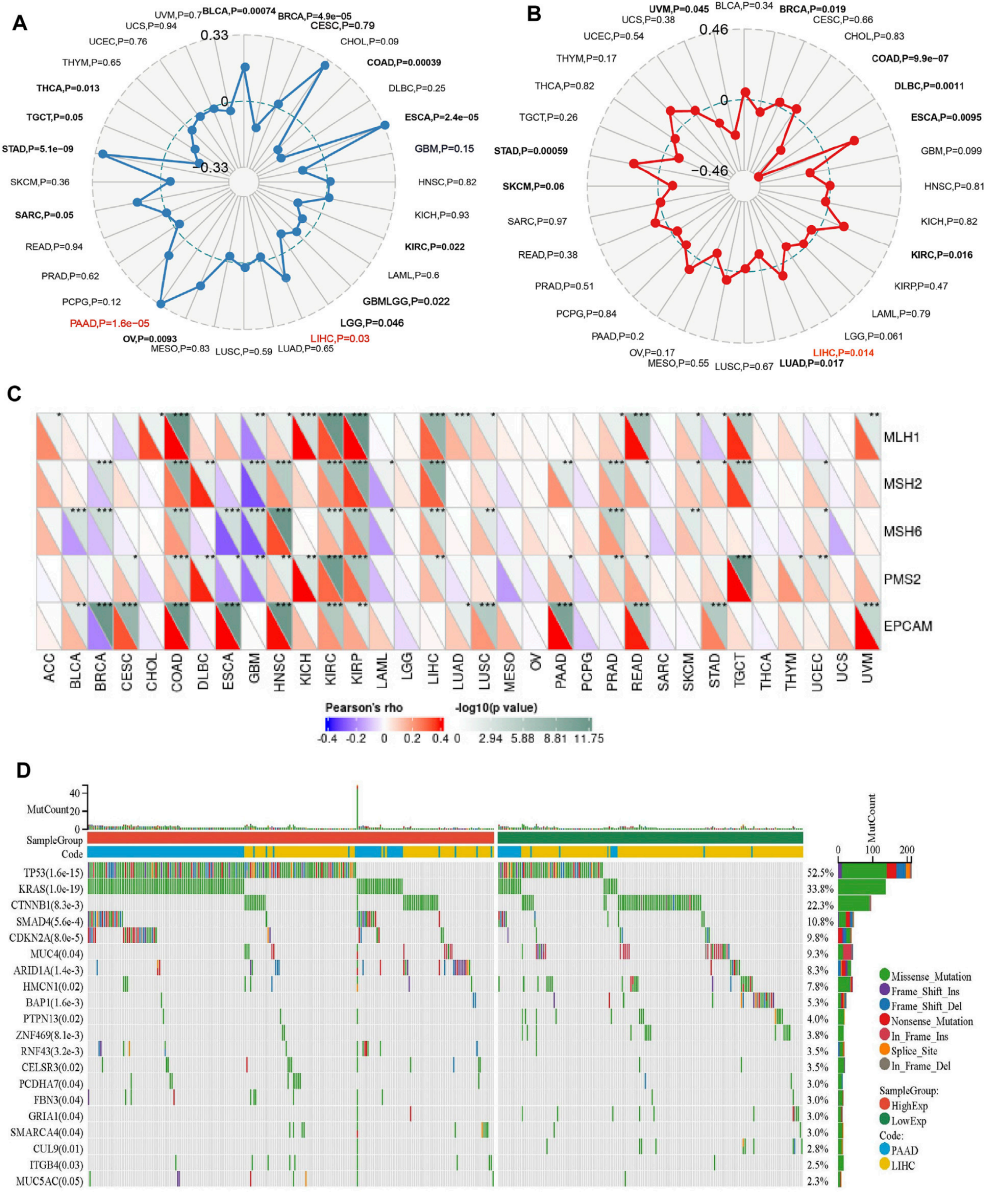
Relationship between PPARG expression and somatic mutation **(A)**, Tumor mutational burden **(B)**, Microsatellite instability **(C)**, and Mismatch repair genes **(D)** in human cancers. (*p < 0.05, **p < 0.01, and ***p < 0.001).

The higher tumor mutational burden (TMB) signifies increased genetic abnormalities and is linked to elevated microsatellite instability (MSI). These alterations enhance tumor cells’ adaptability, potentially leading to treatment evasion and resistance (Petri and Sanz, 2018; Roudko et al., 2020; Fusco et al., 2021). As shown in Figure 5A, the results demonstrate a significant positive correlation between PPARG mRNA expression and TMB in both PAAD and LIHC. This suggests that PPARG is associated with an increased mutational burden in tumors, thereby promoting disease progression. While no statistically significant association was found between the other tumors and MSI, a direct correlation was observed between MSI and PPARG expression in BRAC, COAD, DLBC, ESCA, KIRC, LUAD, SKCM, and STAD. Among the tumors with high PPARG expression and poor prognosis, only LIHC showed a positive correlation between high PPARG expression and MSI (Figure 5B).

Mismatch repair (MMR) genes are essential for maintaining genomic stability and preventing the accumulation of genetic damage. We evaluated the association between mutations and gene expression in five MMR genes: *MLH1, MSH2, MSH6, PMS2,* and *EPCAM*, using data from the TCGA expression profiling project. In PAAD, there was a significant positive association between PPARG expression and the MMR genes *EPCAM, PMS2,* and *MSH2*. However, in LIHC, except for *EPCAM*, there was no significant correlation between PPARG expression and the other MMR genes, indicating a lack of association (Figure 5C). This suggests that PPARG does not directly affect the MMR family genes, thereby not leading to an increase in genomic instability.

In our previous study, we observed a low mutation rate of PPARG in PAAD and LIHC (0.5%) (Figure 4). However, it may still influence cancer progression by affecting key oncogenes or tumor suppressor genes. To further investigate this, we analyzed co-occurring gene mutations associated with PPARG expression. The mutation landscape plot revealed the presence of prominent genes such as *TP53, KRAS, CTNNB1,* and *SMAD4* in LIHC and PAAD, which are known to promote tumor initiation and development (Figure 5D). Mutations in oncogenes not only lead to a loss of response to targeted anticancer therapies but also activate critical signaling pathways and regulatory mechanisms, thereby conferring advantages in tumor proliferation, migration, and invasion.

### 3.7 Alterations in the immune microenvironment induced by dysregulated PPARG in LIHC and PAAD

**FIGURE 6:**
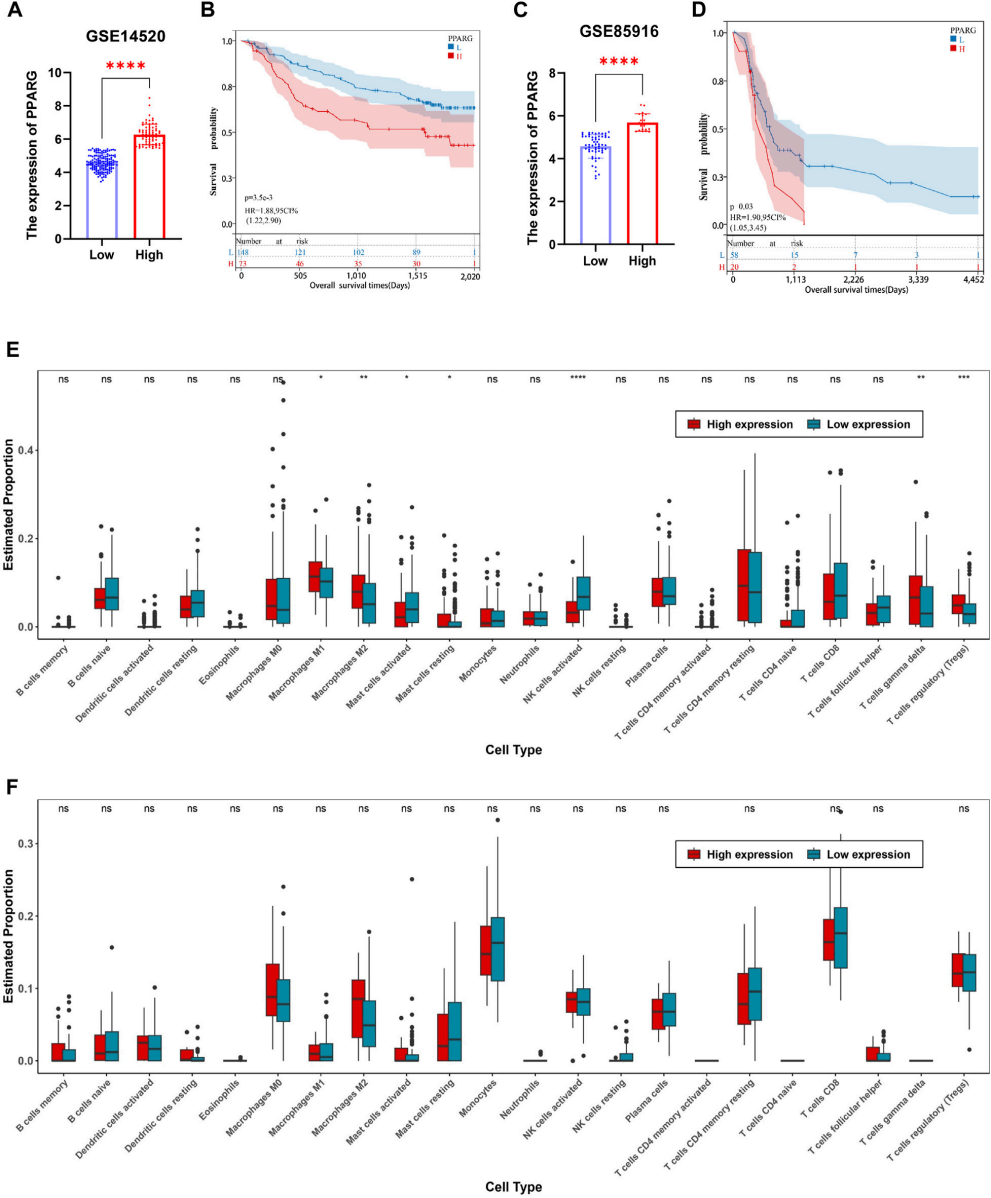
Relationship between PPARG expression and tumor microenvironment in human tumors. **(A)** In accordance with the optimal cutoff value determined using the R software package “maxstat”, the expression of PPARG in the liver cancer dataset GSE14520 was categorized into high-expression and low-expression groups. **(B)** Prognostic survival analysis based on stratification of high and low expression groups of PPARG. **(C)** Stratification of PPARG expression into high and low expression groups in the pancreatic cancer dataset GSE85916 based on the optimal cutoff value determined using the R software package “maxstat”. **(D)** Prognostic survival analysis based on stratification of high and low expression groups of PPARG in the GSE85196 dataset. **(E)** Evaluation of differential immune cell infiltration among high and low expression groups of PPARG using the CIBERSORT algorithm combined with the LM22 signature matrix in the GSE14520 dataset. **(F)** Evaluation of differential immune cell infiltration among high and low expression groups of PPARG using the CIBERSORT algorithm combined with the LM22 signature matrix in the GSE85196 dataset. (*p < 0.05, **p < 0.01, and ***p < 0.001).

The TCGA dataset analysis reveals a significant correlation between PPARG expression and the occurrence of cancer, as well as a poor prognosis. To further investigate this association, we obtained mRNA expression matrices from the GEO database, specifically for LIHC (GSE14520) and pancreatic cancer (GSE85916). These matrices included both tumor and adjacent non-tumor tissues, accompanied by clinical data associated with the samples. Based on these datasets, we calculated the optimal cutoff value for the PPARG expression using the R software package “maxstat.” Subsequently, we categorized patients with tumors from both datasets into two groups: high and low PPARG expression groups. Survival analysis was conducted using the R software package “survival” to assess prognostic differences (Figures 6A, B).

Our findings demonstrate a significant prognostic difference in patients with high PPARG expression levels in both liver and pancreatic cancers (Figures 6C, D). These findings support the notion that higher PPARG expression significantly impacts the prognosis and survival of patients with these malignancies, which aligns with our TCGA database analysis. Furthermore, there is increasing evidence emphasizing the critical role of TME in tumor development. Hence, in this study, we investigated the composition of the TME between the high and low PPARG expression groups. Specifically, using the cell-type identification by estimating relative subsets of RNA transcripts (CIBERSORT) method in conjunction with the LM22 signature matrix, the differential immune cell infiltration was assessed between the high and low PPARG expression groups in the aforementioned GEO datasets.

Our analysis revealed significant differences in immune cell infiltration between the two PPARG expression groups. Specifically, patients with liver cancer with high PPARG expression exhibited a significant increase in the proportions of macrophages M1, macrophages M2, T cells (δ and γ), and T cell regulatory (Tregs). Conversely, the proportions of natural killer (NK) cells activated and mast cells activated were significantly decreased (Figures 6E, F). These findings provide valuable insights into the immune landscape associated with PPARG expression in liver cancer, indicating the potential immunomodulatory effects of PPARG in shaping the TME. Contrastingly, no discernible differences in the immune cell composition were observed between the patients with pancreatic cancer with high and low PPARG expression. Subsequently, we evaluated the association between PPARG gene expression and the statistically significant differences in immune cell populations (Supplementary Figures S7A, 7B). The results revealed a positive correlation between PPARG and macrophages M2, T cell regulatory (Tregs), and T cell (δ and γ), while showing a negative correlation with NK and mast cells activated (Supplementary Figures S7C–7G). These findings suggest that the association between PPARG expression and immune cell infiltration may vary across various types of cancer.

While immune activation and infiltration are crucial for tumor suppression, tumor cells often exploit excessive immune responses and infiltration to establish immune evasion mechanisms. Excessive immune infiltration can lead to immune dysfunction, weakening the anticancer effect and simultaneously promoting tumor progression and dissemination. Elevated ICP expression levels are frequently associated with cancer progression and may contribute to immune evasion, drug resistance, and an unfavorable prognosis in tumor development. In this study, we collected ICP genes and analyzed their correlation with PPARG gene expression. A Pearson correlation analysis was performed to assess the association between PPARG and ICP genes. The results showed a significant positive correlation between PPARG and ICPs in LIHC, in contrast to PAAD. These findings suggest that PPARG effectively regulates ICP expression to evade immune attack (Supplementary Figure S8).

### 3.8 Correlation between PPARG expression and sensitivity spectrum to anticancer drugs and molecular docking simulation validation

**FIGURE 7:**
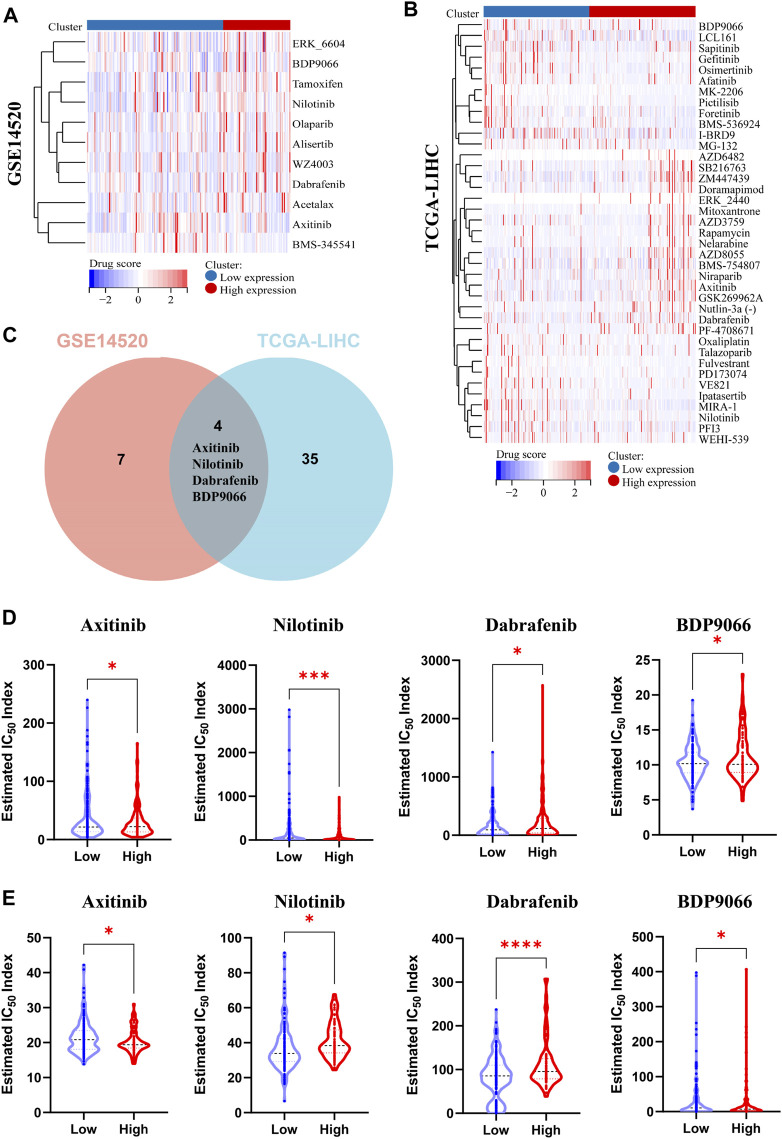
Analysis of multiple datasets revealed the relationship between PPARG expression and drug sensitivity profiles. **(A)** The OncoPredict algorithm was used to calculate the drug sensitivity of the liver cancer dataset GSE14520. **(B)** The OncoPredict algorithm was employed to calculate the drug sensitivity of liver cancer datasets from the TCGA-LIHC database. **(C)** The Venn diagram was used to identify the drugs selected by fitting two datasets. **(D)** The relationship between PPARG expression and the sensitivity of four drugs, namely, Axitinib, Nilotinib, Dabrafenib, and BDP9066, in the TCGA-LIHC dataset. **(E)** The association between PPARG expression and drug sensitivity of the same four drugs in the GSE14520 dataset. (*p < 0.05, **p < 0.01, and ***p < 0.001).

Gene expression plays a crucial role in driving molecular and epigenetic changes within the human body, influencing individual responses to disease treatment drugs, and altering drug sensitivity. In this study, we further investigated the differences in drug sensitivity between patients with high and low PPARG expression levels using RNA-sequencing data. Through genomic stability and immune microenvironment analysis, we identified a significant association between PPARG expression and these factors in LIHC. Subsequently, we evaluated the drug sensitivity profiles in the GSE14520 dataset (LIHC) from the GEO database and the TCGA-LIHC patient cohort from the TCGA database using the OncoPredict method (Figures 7A, B). The findings revealed significant differences in drug sensitivity between the groups with high and low PPARG expression. These findings highlight the potential impact of PPARG expression on drug response and emphasize its relevance in personalized medicine for the treatment of cancer. Furthermore, by integrating drug sensitivity profiles from the TCGA-LIHC and GSE14520 datasets, we identified a common set of drugs, including Nilotinib, BDP9066, Dabrafenib, and Axitinib (Figure 7C). Notably, all four drugs are targeted therapies for the treatment of cancer. The drug sensitivity analysis revealed that patients with high PPARG expression in TCGA-LIHC and GSE14520 may exhibit increased sensitivity to Axitinib and Nilotinib (Figures 7D, E).

**FIGURE 8:**
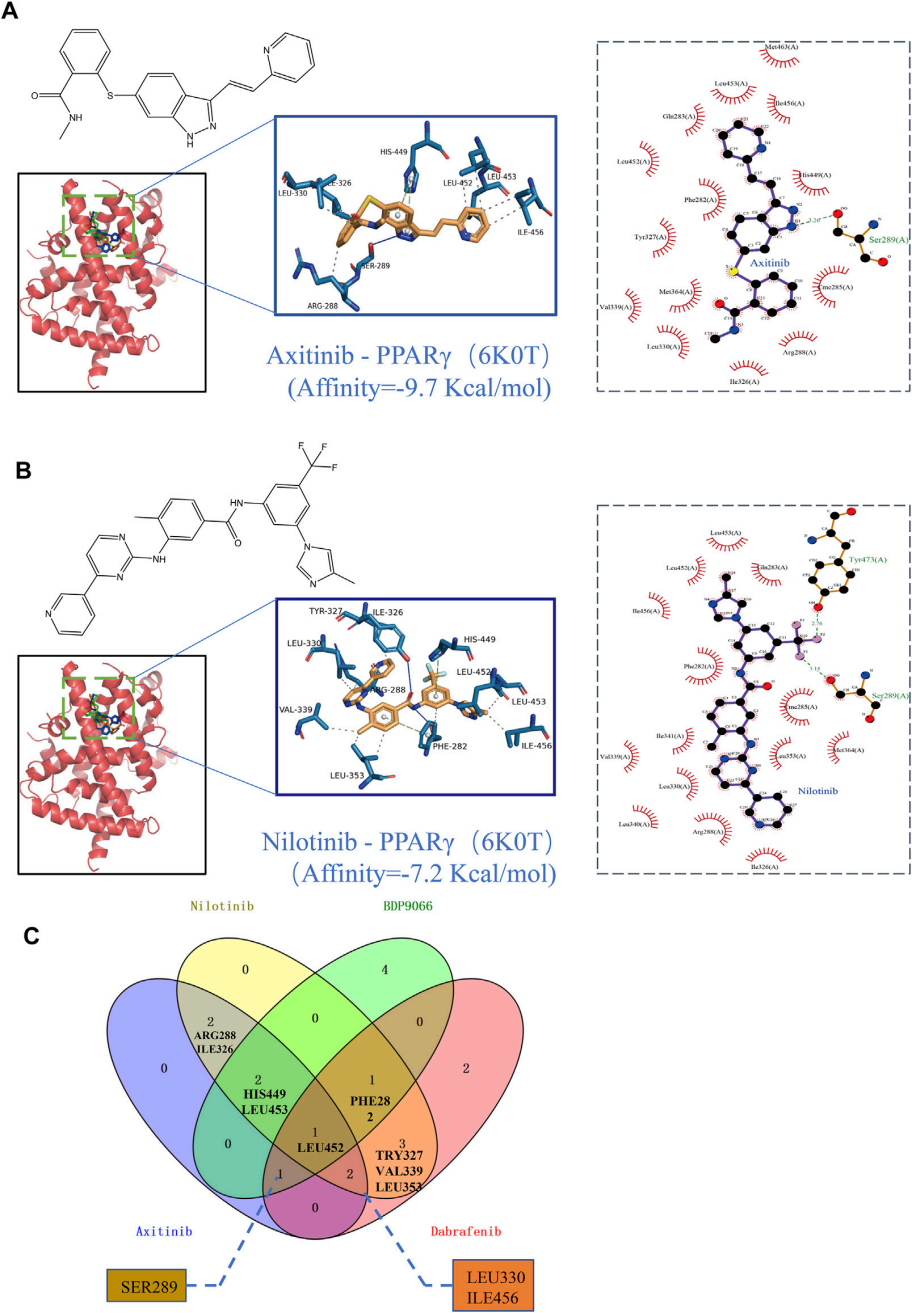
Binding scores and binding conformations of potential therapeutic drugs with PPARγ (PDB: 6K0T) simulated using Autodock Vina. **(A)** Analysis of the two-dimensional binding mode of Nilotinib with 6K0T using LigPlot + software (left panel) and the three-dimensional binding conformation using Pymol (right panel), with a binding score of −9.7 kcal/mol. **(B)** Analysis of the two-dimensional binding mode of Axitinib with 6K0T using LigPlot + software (left panel) and analysis of their three-dimensional binding conformation using Pymol (right panel), with a binding score of −7.2 kcal/mol. **(C)** Venn chart analysis of key amino acids in the active pocket for four small molecule anticancer drugs.

The PPARgama molecular pocket formation and its binding with ligands are crucial for the anti-proliferative activity against tumors (Yamamoto et al., 2019). Molecular docking was employed to validate the potential binding of Nilotinib, Axitinib, Dabrafenib, and BDP9066 with the active pocket of PPARG. Our results demonstrated robust binding modes and remarkably low binding energies, indicating stable and favorable interactions between these four anticancer drugs and PPARG (Figures 8A, B). Notably, Axitinib, BDP9066, Dabrafenib, and Nilotinib exhibited notable molecular docking capabilities, with affinity values of −9.7, −6.7, −9.6, and −7.2 kcal/mol, respectively, as determined by the absolute values of their binding energies (Supplementary Figures S9A, 9B). Interactions between four small-molecule anticancer drugs and key amino acid residues within the active pocket were investigated by Venn diagram analysis to examine the molecular interactions and binding affinity of these small molecules with 6K0T (Figure 8C). This analysis illustrates the statistical distribution and frequency of these key amino acids within the active pocket, shedding light on the molecular interactions and binding affinity of the small-molecule drugs. These findings offer potential mechanisms of action for inhibiting tumor growth.

### 3.9 Inhibition of hepatocellular carcinoma cell proliferation activity through selective targeting of PPARγ

**FIGURE 9:**
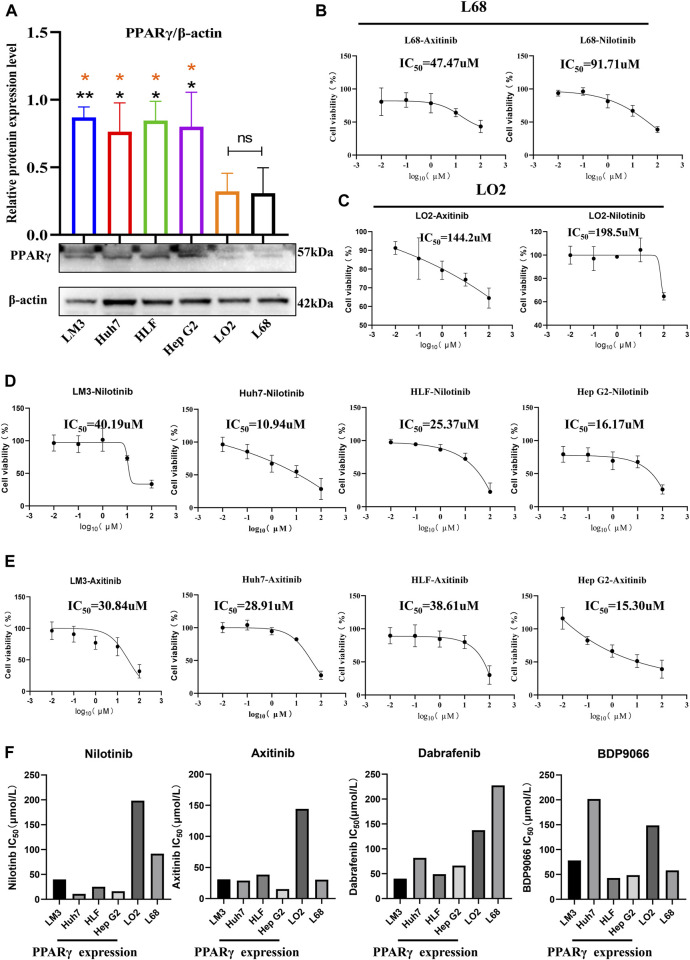
Relationship between the expression of PPARγ and drug sensitivity.**(A)** Expression of PPARγ in four hepatocellular carcinoma cell lines (LM3, Huh7, HLF, and Hep G2) and two control liver cell lines (LO2 and L68). **(B,C)** Drug Sensitivity of Nilotinib and Axitinib in control Hepatic Cells LO2 and L68. **(D,E)** Drug sensitivity of Nilotinib and Axitinib in four Hepatocellular Carcinoma Cell Lines LM3, Huh7, HLF, and Hep G2. **(F)** Bar graph of PPARγ expression levels and drug sensitivity of four small molecule compounds.

In examining PPARγ expression between cancer cells and healthy control cells, we observed significantly higher PPARγ expression in the four hepatocellular carcinoma cell lines (LM3, HLF, Huh7, and Hep G2) compared to the two normal liver cell lines (LO2 and L68) (Figure 9A). Subsequently, cell proliferation assays were conducted after incubating the 6 cell lines with the four drugs at various concentrations for 24 h. The results revealed increased sensitivity to all four drugs with high PPARγ expression. Particularly, Nilotinib and Axitinib demonstrated significant anti-proliferative activity against liver cancer cells. In Huh7 cells, Nilotinib exhibited an IC_50_ of 10.94 uM, while in Hep G2 cells, the IC_50_ remained around 15 uM. For LM3 cells, both drugs maintained IC_50_ values between 30 and 40 uM. Conversely, Dabrafenib showed weaker inhibitory effects on liver cancer cell proliferation compared to the aforementioned two drugs, with the highest IC_50_ reaching 81.86 uM. However, even in LM3 cells, it reached 40.19 uM (Supplementary Figure S10). Notably, the inhibitory effects of the four drugs on normal liver cells were consistently lower compared to their effects on liver cancer cells (Figures 9D, E). Axitinib exhibited an IC_50_ of 144.2 uM in LO2 cells, while Nilotinib reached concentrations close to 200 uM. Similarly, Dabrafenib demonstrated a similar pattern of activity (Figures 9B, C). Furthermore, we noted a correlation between the four drugs’ inhibitory effects on cell proliferation and PPARγ expression. Cells with elevated PPARγ expression in liver cancer displayed increased drug sensitivity (Figure 9F). The results were consistent with the predictions from the Oncopredict algorithm and the protein level validation. Through predictive analysis and experimental testing, we confirmed its potential as a target for cancer treatment.

### 3.10 The co-expression networks of PPARG demonstrate associations with relevant molecular mechanisms

**FIGURE 10:**
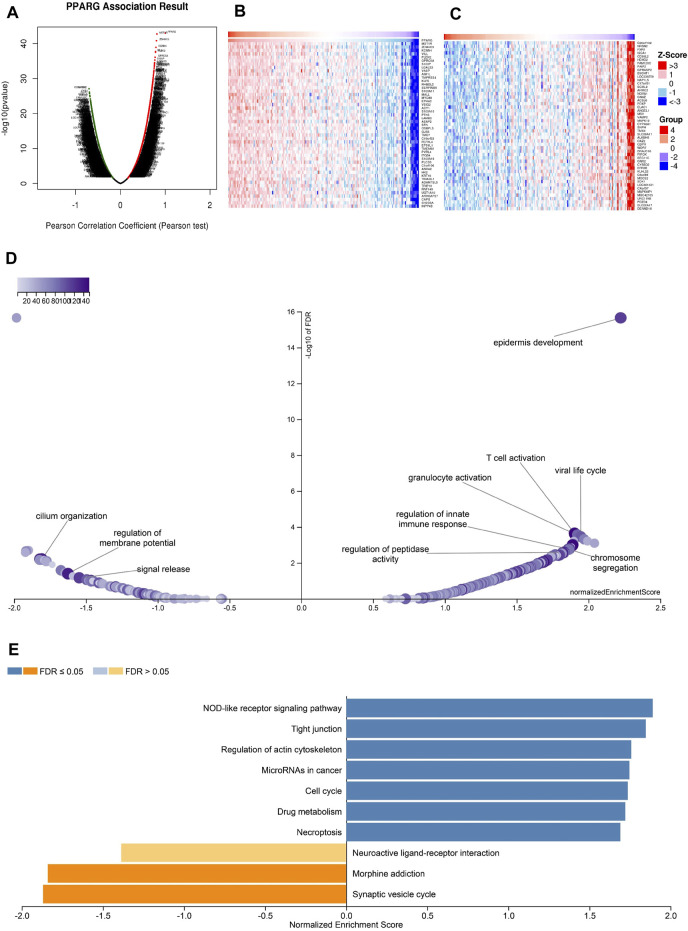
PPARG co-expression genes in LIHC analyzed by the LinkedOmics database. **(A)** Highly relevant genes for PPARG expression in the LIHC cohort examined by Pearson’s test. Top 50 positive co-expression genes **(B)** and negative co-expression genes **(C)** of in heat map in LIHC. **(D)** Volcano plot of PPARG KEGG pathways in LIHC cohort. **(E)** Directed acyclic graph of PPARG GO analysis (biological process) in LIHC cohort.

The above findings further validate the prognostic value of PPARG in pan-cancer and its significant association with the immune response. Elevated PPARG expression in LIHC and PAAD is significantly positively correlated with poor OS, DSS, PFI, and DFI (Figure 2; Supplementary Figure S1). To validate the potential role of the PPARG gene as a biomarker in pan-cancer, its functions and heterogeneity, as well as its potential role in LIHC, were investigated. The LinkOmics database PPARG co-expression network was used for this analysis (Supplementary Figure S11). In LIHC, there was a significant positive correlation between PPARG expression and 4,820 genes (represented by dark red dots) as well as a significant negative correlation between 5,775 genes (represented by dark green dots) [false discovery rate (FDR) <0.01] (Figure 10A). The top 50 genes that exhibited the strongest correlation with PPARG expression are shown in Figure 10B, while Figure 10C shows the top 50 genes with the strongest negative correlation. Furthermore, the positive and negative correlations of the top 50 genes co-expressed with PPARG are shown in Supplementary Tables S1, S2, respectively. Notably, *MST1R, ZDHHC3,* and *KCNN* emerged as the top three genes exhibiting the most significant correlation with PPARG co-expression.

Further investigation of GO BP categories revealed the involvement of PPARG and its co-expressed genes in various functions, including T cell activation, immune response control, and regulation of peptidase activity. This determination was made by using GSEA to identify the major GO terms enriched among PPARG-co-expressed genes (Figure 10D). The co-expressed genes were enriched in the nucleotide oligomerization domain (NOD)-like receptor signaling pathway, tight junction, control of the actin cytoskeleton, cell cycle, and drug metabolism, according to a KEGG pathway analysis (Figure 10E). These findings imply that PPARG expression may influence various human cancers by affecting the immune response within TME.

## 4 Discussion

PPARG acts as a nuclear receptor that binds peroxisome proliferators, including lipid-lowering drugs and fatty acids. Upon activation by the ligand, the nuclear receptor binds to DNA-specific PPAR response elements (PPRE) and regulates the transcription of its target genes (Park et al., 2010). One such target gene is acyl-coenzyme A oxidase, which controls the peroxisomal β-oxidation pathway of fatty acids. PPARG plays a crucial role as a key regulator of adipocyte differentiation and glucose homeostasis (Mukherjee et al., 1997). While significant progress has been made in understanding the biological activities and functions of various molecules through advancements in molecular biology, there is still a need for further exploration of their roles in human cancer and their potential as therapeutic targets. Previous studies have shown that PPARG gene expression levels are significantly upregulated in bladder cancer, esophageal adenocarcinoma, and prostate cancer. Moreover, they have been associated with treatment resistance and a poor prognosis in cancer (Galbraith et al., 2018; Liu et al., 2019; Ma et al., 2021). In bladder cancer, the potential of PPARG as a biomarker has been confirmed (Chiu et al., 2017). Furthermore, previous studies have demonstrated a significant association between high PPARG expression and poor prognosis in pancreatic ductal adenocarcinoma, with two key contributing factors being obesity and diabetes (Andersen et al., 2017). These findings align with our current research findings. Concerning lung cancer, miR-130a and miR-130b have been identified to play a regulatory role in macrophage polarization by inhibiting or downregulating PPARG expression (Lin et al., 2015). Moreover, they stimulate vascular endothelial growth factor A (VEGF-A) and anti-apoptotic factor (B-cell lymphoma-2, Bcl-2) expression (Tian et al., 2022), thereby influencing the progression of lung cancer. The PPARG differential expression in human cancers is often associated with tumor growth. However, there remains uncertainty regarding the precise procedures and pathways through which PPARG influences tumor development, leading to a poor prognosis.

Through our investigation, we confirmed the variability of PPARG expression in various types of cancers by analyzing PPARG gene expression levels in human cancers using the TCGA database. Subsequently, we examined the association between PPARG expression heterogeneity and survival outcomes, finding that high PPARG expression in various types of cancer was strongly associated with a poor prognosis. This suggests that the upregulation of PPARG may contribute to human cancer development, particularly in these specific types of cancer. Additionally, our PPARG gene alteration analysis revealed a mutation frequency of 0.5%. Furthermore, we observed higher rates of mutations, including missense mutations, truncating mutations, amplifications, and profound deletions, in PAAD. These findings indicate a potential role in tumorigenesis and cancer progression. The correlation between PPARG and TMB, MSI, and MMRs also demonstrated a close association between PPARG and TME in human cancers.

Furthermore, our findings using immunohistochemical experiments conducted on pathological sections of patients with LIHC and PPAD, where PPARG expression was significantly elevated, validated our previous analysis. These results demonstrated a strong association between PPARG overexpression, tumorigenesis, and cancer progression. Hence, PPARG has the potential to serve as a molecular marker for cancer. Consequently, an *in vitro* screening of anticancer drugs targeting PPARG was performed, which validated our findings. The results revealed a positive correlation between the drug sensitivity of these drug categories and PPARG expression. However, it was observed that BDP9066 exhibited lower selectivity in its anticancer proliferative activity compared to the other three drugs, with an IC_50_ as high as 201.8 µM against Huh7 liver cancer cells. This could be attributed to the binding mode of the small molecule with PPARG and its high binding energy requirement (affinity = −6.7 kcal/mol). To address this issue, further expansion of molecular library screening can be pursued by using ligand- or receptor-based computationally-assisted drug selection. This approach aims to identify small-molecule compounds with enhanced targeting capabilities. Additionally, conducting high-throughput simulations of PPARG ligand efficiency and elucidating key amino acid residues will facilitate precise drug targeting, thereby reducing off-target effects and minimizing the potential for low anticancer activity and high toxic side effects. These advancements will facilitate the clinical application of targeted therapies directed at the potential biomarker PPARG. Furthermore, to understand its potential mode of action, we examined PPARG expression in several immunological and molecular subtypes of human malignancies. The findings revealed significant variations in PPARG expression among different immunological and molecular subgroups in LIHC.

Immune infiltration is typically associated with the suppression of tumor growth and metastasis. However, when immune infiltration becomes excessive, it enables tumor cells to evade immune attacks. Tumor cells establish immune escape mechanisms through various means, including inhibitory factors and ICP molecule expressions such as programmed death-1 (PD-1) and programmed death ligand-1 (PDL-1) (Diesendruck and Benhar, 2017; Sharma et al., 2023). According to this study, increased PPARG expression is associated with a poor prognosis. Notably, PPARG expression is associated with the expression of several ICPs in LIHC. Furthermore, excessive immune infiltration can lead to functional abnormalities within TME.

Recent research has highlighted the involvement of PPARG in regulating lipid degeneration and promoting neutrophil infiltration in the liver during the transition from inflammatory liver disease and fatty liver to hepatocellular carcinoma (Chan et al., 2017; Wang et al., 2017). The attachment of neutrophils to the luminal surface of blood vessels and their subsequent migration to the site of the lesion outside the blood vessels relies on the activation of integrins by chemotactic proteins (Mohs et al., 2017; Wu et al., 2021). Neutrophils play a critical role in the primary defense against infections and tissue damage, and they also exhibit immunoregulatory functions. However, the recruitment of a significant number of neutrophils by chemotactic factors can have detrimental effects. Persistently high expression or upregulation of chemotactic factors, as a part of the active physiological feedback mechanism of the body, can exacerbate the degradation of the extracellular matrix and lead to widespread tissue damage. Consequently, this process directly enhances the survival, invasion, and metastasis capabilities of tumor cells (Hughes and Nibbs, 2018; Petri and Sanz, 2018). Paradoxically, the ultimate outcome can compromise the protective role of immune cells, thus increasing the risk of tissue damage and cancer development.

Moreover, the interaction between PPARG expression and multiple signaling pathways suggests its involvement in modulating the immune microenvironment. These pathways include the NOD-like receptor signaling pathway, T cell activation, cytoskeletal regulation, and drug metabolism. Notably, the NOD-like receptors have been identified as crucial factors in tumorigenesis, angiogenesis, cancer stemness, and chemoresistance. In endometrial cancer (EMC), dysregulation of the NOD-like receptor family protein (NLRP) has been shown to impair the phagocytic function of macrophages and reduce the CD + T cell population, thereby promoting the growth, invasion, and metastasis of EMC cells (Zhu et al., 2023). Furthermore, recent research has revealed that tumor cells can utilize lipids secreted by adipocytes to promote tumor progression and chemoresistance through metabolic reprogramming. In ovarian cancer, adipocytes, as a significant component of the ovarian TME, impact drug distribution and contribute to chemoresistance (Yang et al., 2019). Considering PPARG as a key regulator of adipogenesis and differentiation, it may be one of the underlying contributing factors to tumor progression and chemoresistance. Thus, the manipulation of PPARG expression holds promise as a potential strategy for shaping the immune microenvironment and developing novel therapeutic approaches to combat tumor growth and metastasis.

In summary, the pan-cancer analysis and experimental investigations of PPARG have confirmed its differential expression across various types of cancers and its association with survival outcomes and poor prognosis, including in PAAD, LIHC, LGG, GBM, and other types of cancer. These associations have been attributed to PPARG gene variants, epigenetic modifications such as acetylation and phosphorylation patterns, and effects on immune infiltration. However, despite the comprehensive research on PPARG, there are certain limitations to this study. Firstly, more direct clinical studies are required to further elucidate the precise function of PPARG in regulating immunological processes. Secondly, the precise significance of PPARG expression in various types of cancer and its precise role in immune regulation within these tumors remain unclear.

## 5 Conclusion

In this study, we observed heterogeneous PPARG mRNA expression levels across various types of cancer. Moreover, we found a strong correlation between PPARG expression and several important factors, including prognostic value, features related to clinical stages, drug sensitivity profiles, and activation of immune cells. Additionally, we identified PPARG as a key molecule involved in the regulation of key pathways such as the NOD-like receptor signaling pathway, cell cycle, and drug metabolism. These findings highlight the potential utility of PPARG as a valuable cancer biomarker.

## Data Availability

The datasets presented in this study can be found in online repositories. The names of the repository/repositories and accession number(s) can be found below: GSE14520; GSE85916.
