# Supplementary material for: Integrated investigation and experimental validation of PPARG as an oncogenic driver: implications for prognostic assessment and therapeutic targeting in hepatocellular carcinoma
Source: Front Pharmacol. 2023 Nov 15;14:1298341. doi: 10.3389/fphar.2023.1298341 (PMC10690586; doi:10.3389/fphar.2023.1298341)
Supplement: Supplementary file 1 [file DataSheet1.PDF]

Supplementary Material

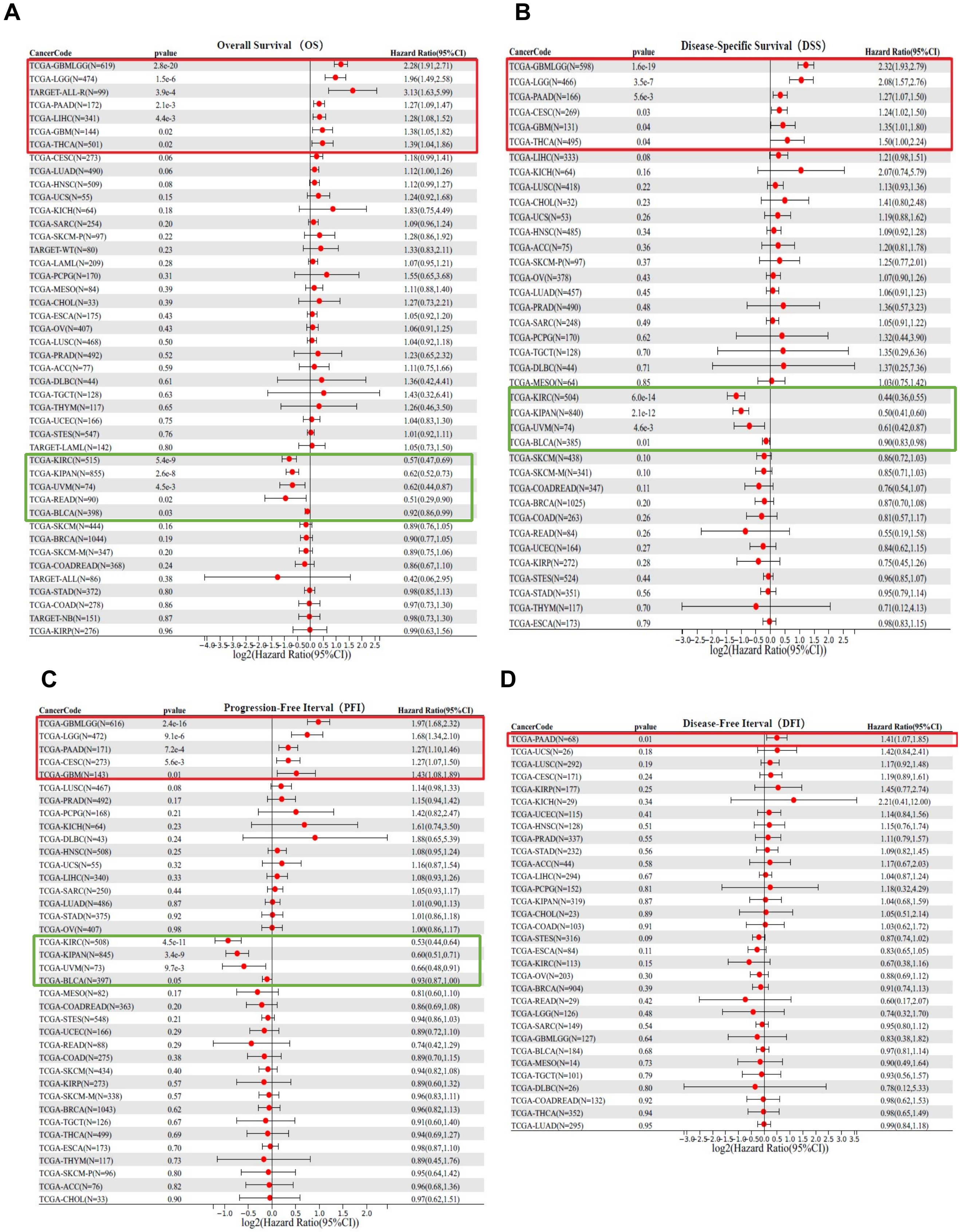

Supplementary Figure 1. Prognostic value of PPARG in various cancers. (A) Forest plot of the association between PPARG expression and OS (A), DSS (B), PFI (C), and DFI (D), in 33 human cancer types.

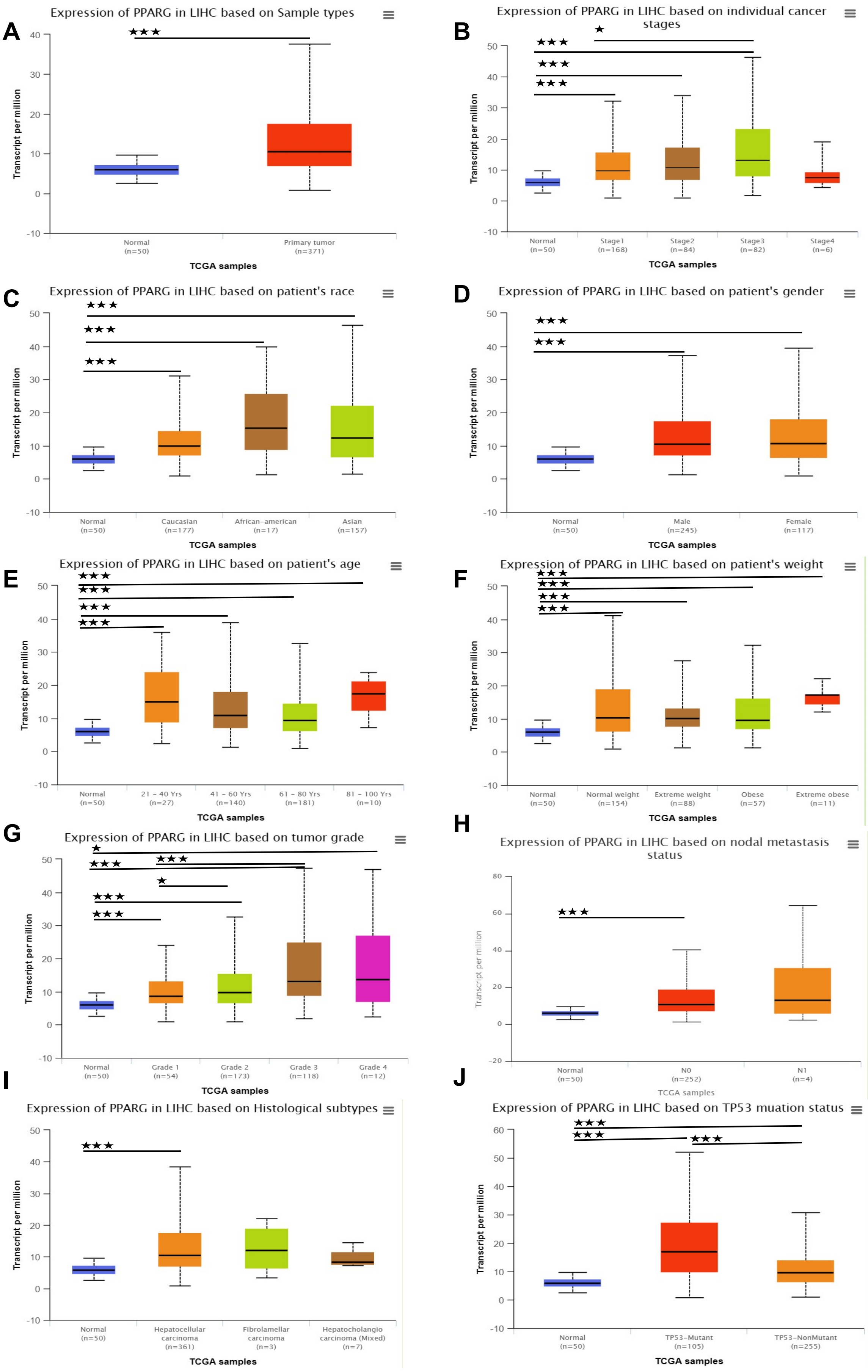

Supplementary Figure 2. Analysis of PPARG genomic alterations in human LIHC through the UALCAN online database. (A-J) Differential expression of PPARG in PAAD according to sample types (n = 421) (A), individual cancer (B) (n = 390) , race(C) (n = 401), gender(n = 412)(D), age(n = 408) (E), weight(n = 360) (F), tumor grade(n = 407) (G), nodal

metastasis(n=306) (H), Histological subtypes(n=421) (I), and TP53 mutation status (n = 410) (J). (*$p < 0.05$, **$p < 0.01$, and ***$p < 0.001$).

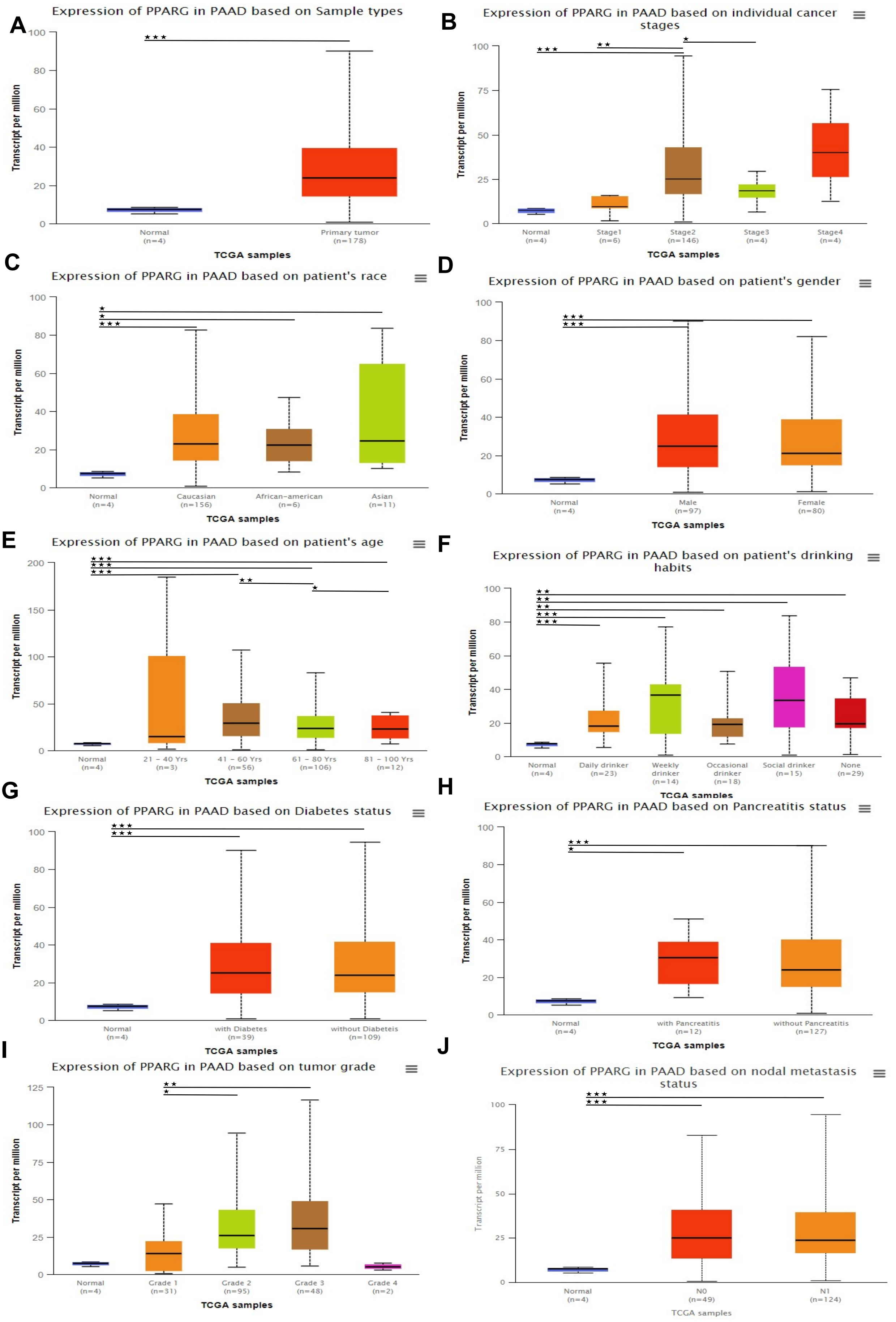

Supplementary Figure 3. Analysis of PPARG genomic alterations in human PAAD through the UNLCAN online database. (A-J) Differential expression of PPARG in PAAD according to sample types (n = 181) (A), individual (B) (n = 164) , race(C) (n = 178), gender(n = 181) (D), age(n = 181) (E), drinking habits(n = 103)(F), Diabetes status (n = 152) (G), pancreatitis status(n = 143) (H), tumor grade(n = 180) (I), nodal metastasis(n = 177) (J), and TP53 mutation status (n = 179) (K). (*$p < 0.05$, **$p < 0.01$, and ***$p < 0.001$).

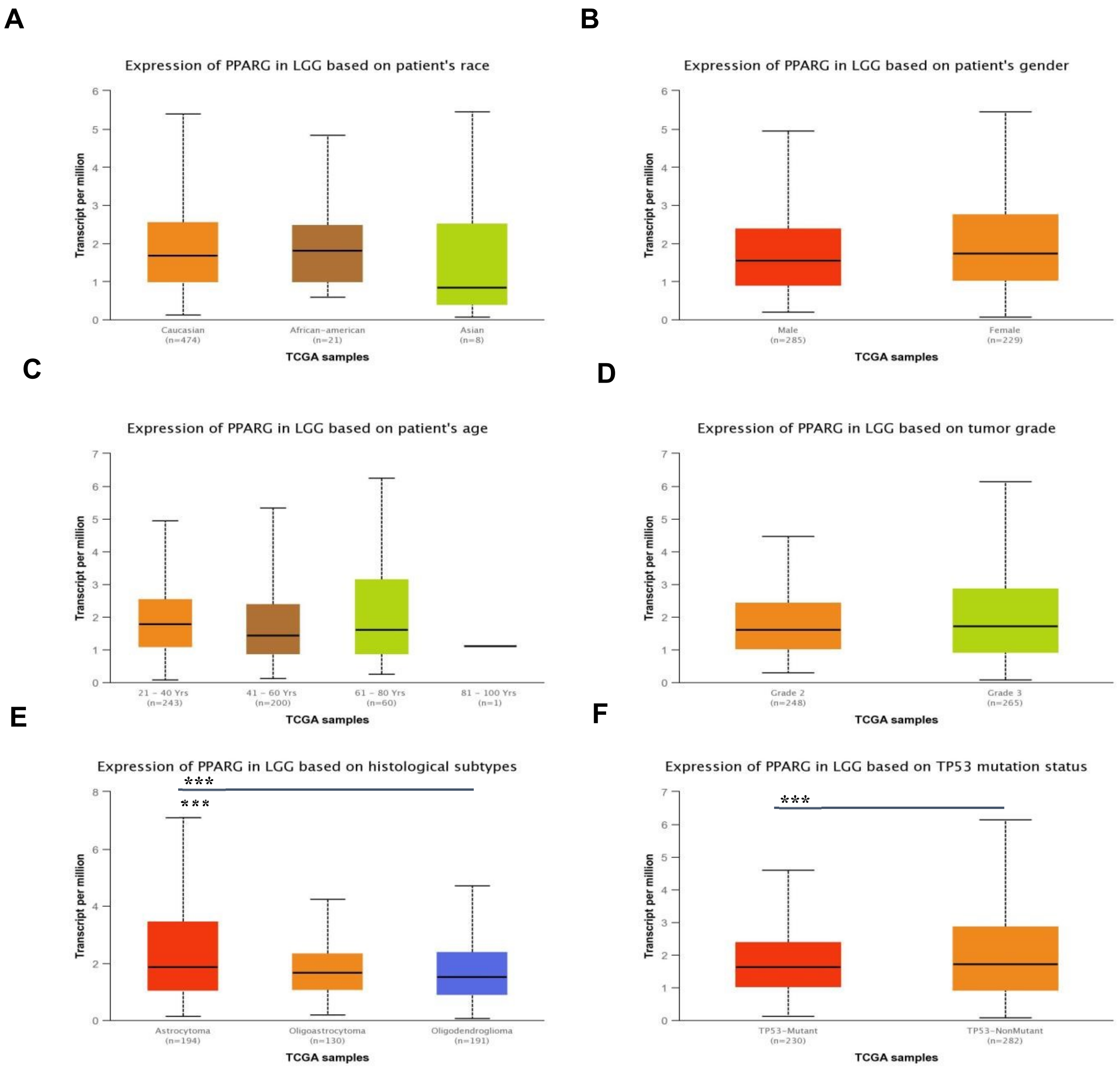

Supplementary Figure 4. Analysis of PPARG genomic alterations in human LGG through the UALCAN online database. (A-F) Differential expression of PPARG in PAAD according to patient's race (n = 503) (A), patient's gender (B) (n = 514) , age(C) (n = 504), tumor grade(n = 513) (D), histological subtypes(n = 513) (E), and TP53 mutation status (n = 512) (J). (*$p < 0.05$, **$p < 0.01$, and ***$p < 0.001$).

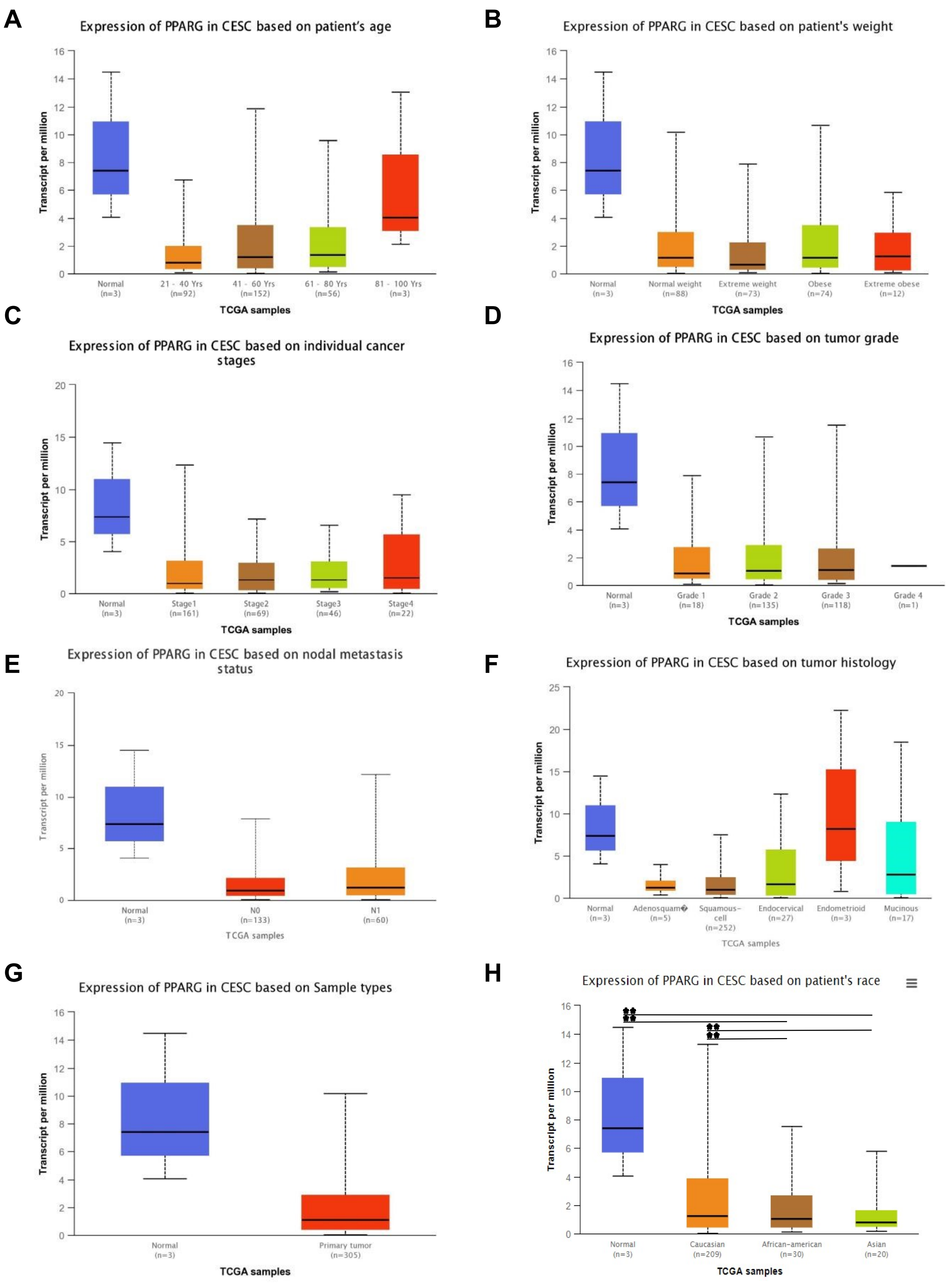

Supplementary Figure 5. Analysis of PPARG genomic alterations in human CESC through the UALCAN online database. (A-H) Differential expression of PPARG in PAAD according to patient's age(n = 301) (A), weight (B) (n = 250), individual cancer stages(C) (n = 301),tumor grade (D) (n = 275) , nodal metastasis(E) (n = 196), tumor histology(F) (n = 317) , sample types(G) (n = 308) and race (H) (n = 262). (**p* < 0.05, ***p* < 0.01, and ****p* < 0.001).

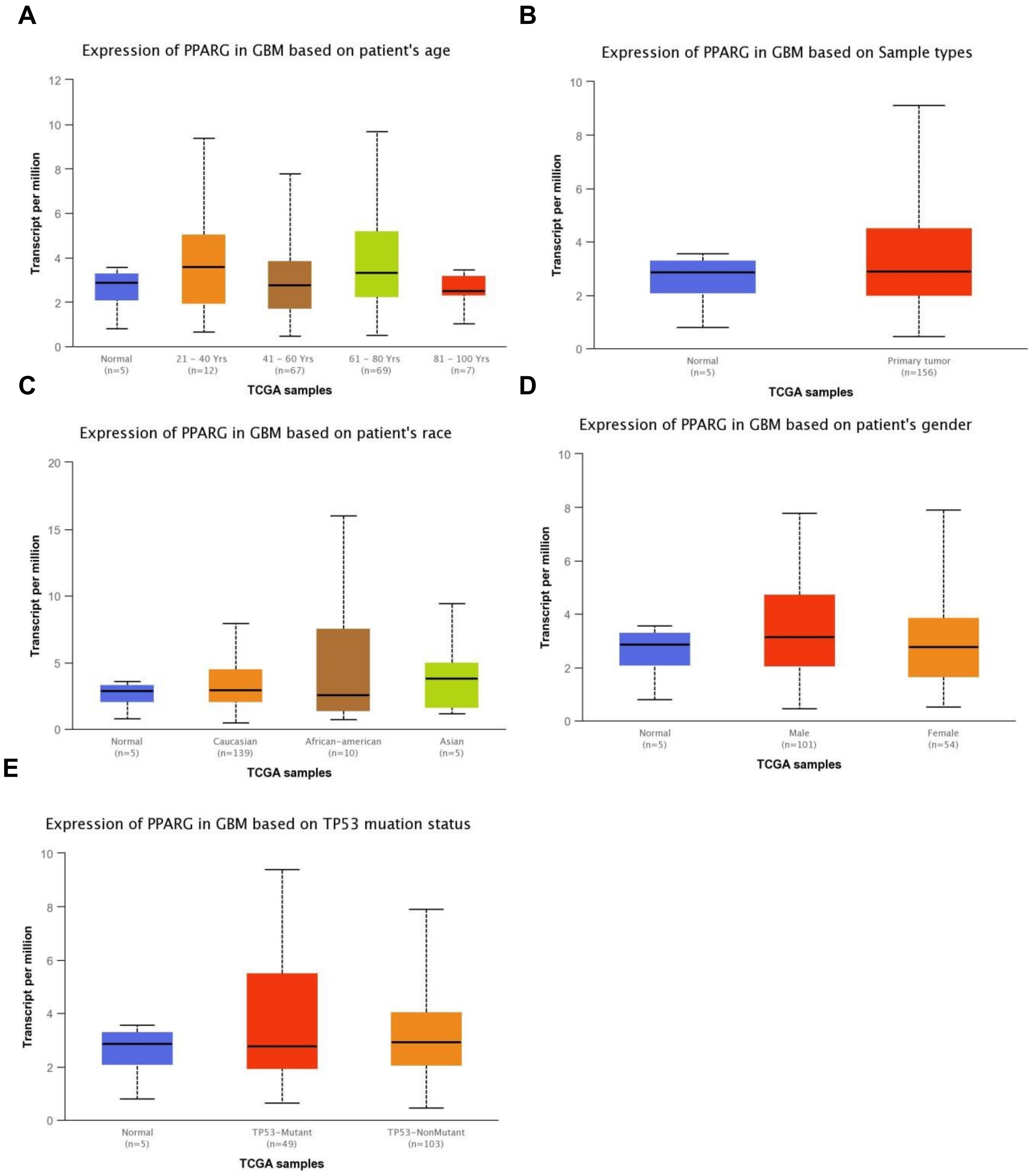

Supplementary Figure 6. Analysis of PPARG genomic alterations in human GBM through the UALCAN online database. (A-E) Differential expression of PPARG in PAAD according to patient's age(n = 160) (A), sample types(B) (n = 161) , race(C) (n = 159), gender(D) (n = 160) , and TP53 mutation (E) (n = 157). (*$p < 0.05$, **$p < 0.01$, and ***$p < 0.001$).

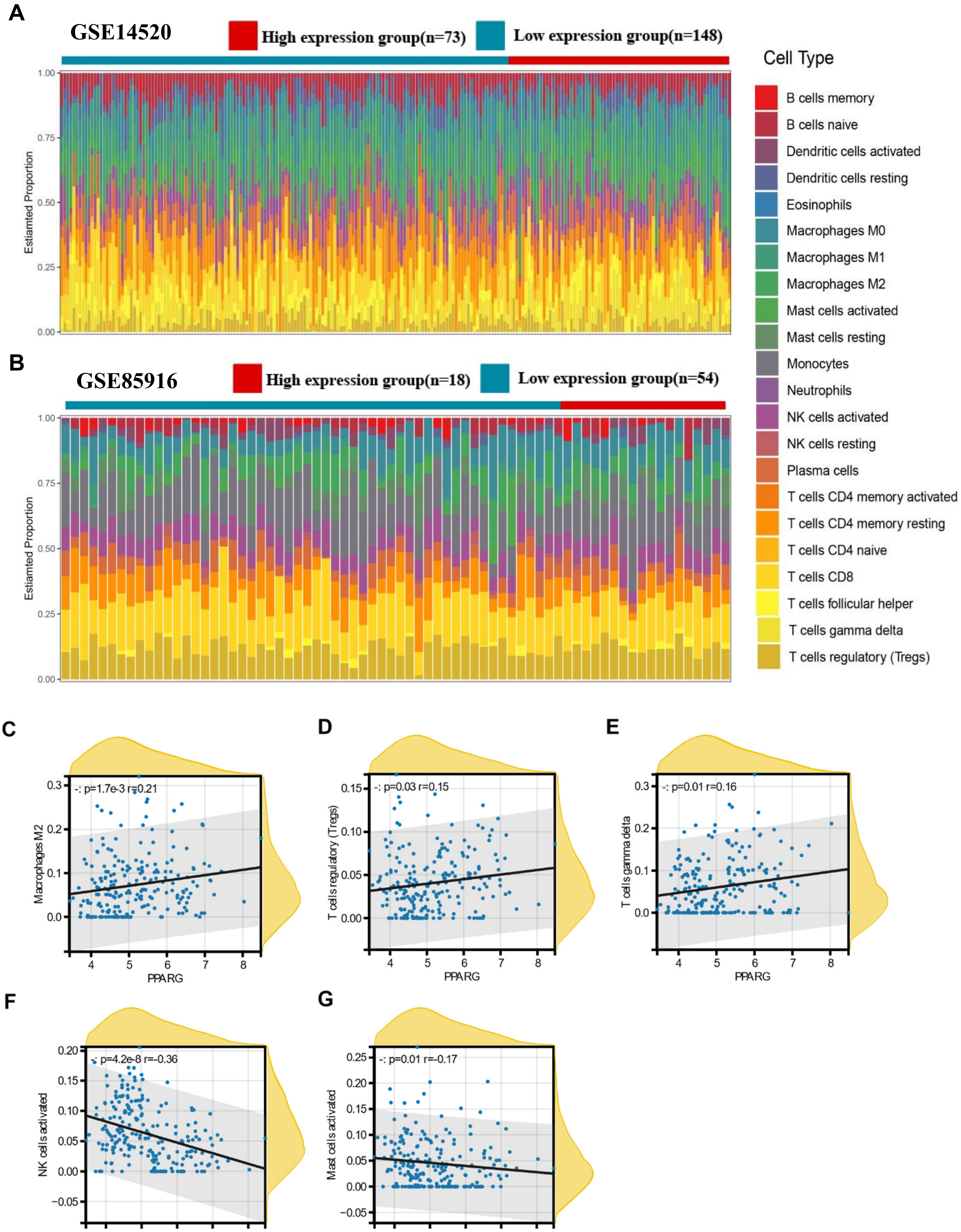

Supplementary Figure 7 Relationship between PPARG expression and tumor microenvironment in human tumors. (A-B) Differential infiltration of 22 immune cell types associated with the expression of PPARG in the GSE14520 and GSE85196 datasets. (C-E) In the GSE14520 dataset, PPARG exhibits a positive correlation with Macrophages M2, T cell

regulatory (Tregs), and T cell gamma delta. (F-G) In the GSE14520 dataset, a negative correlation between PPARG and NK cells activated and Mast cells activated. (*$p < 0.05$, **$p < 0.01$, and ***$p < 0.001$).

A

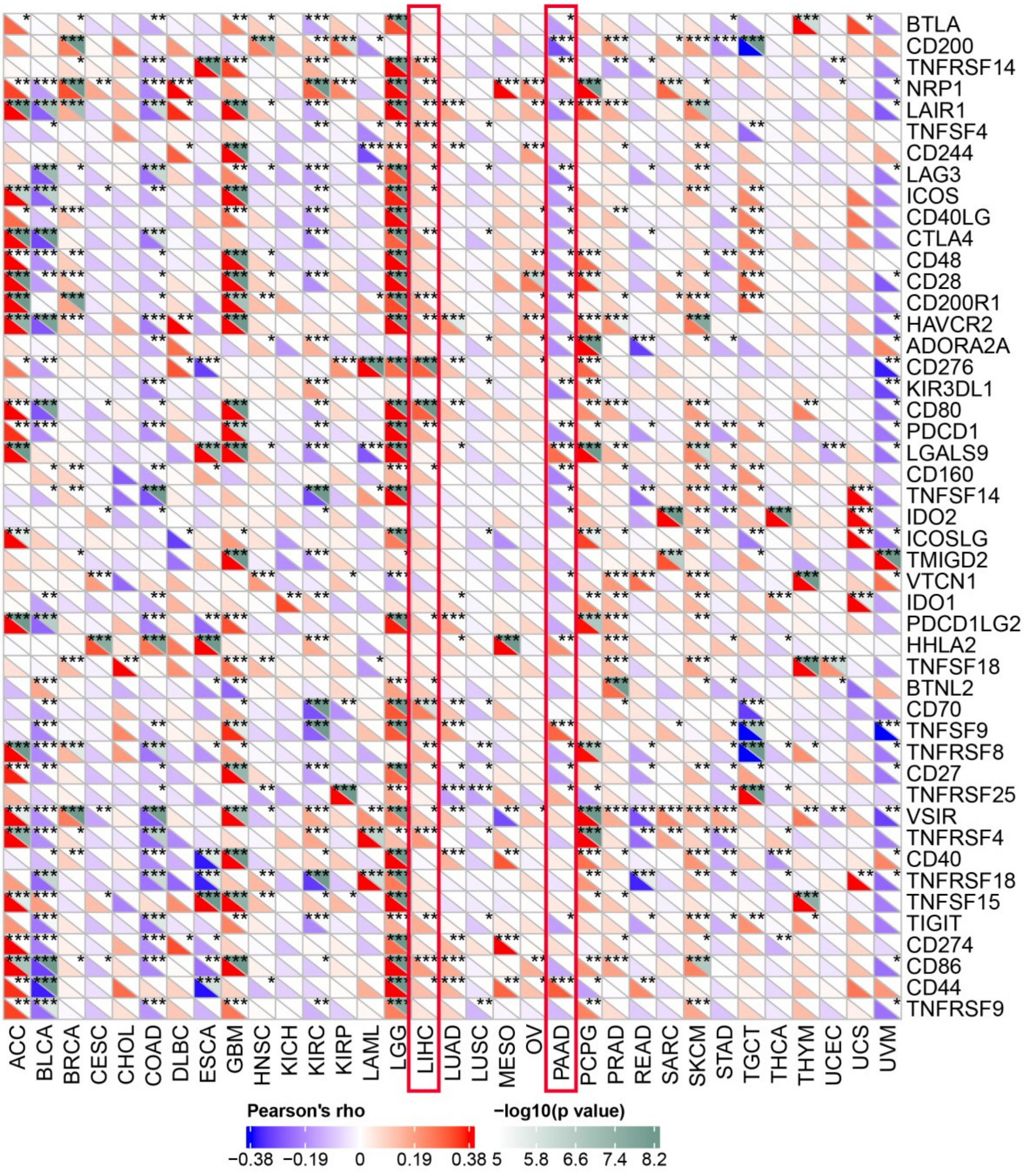

Supplementary Figure 8. The relationship between PPARγ expression and pan-cancer immune checkpoint genes. (*$p < 0.05$, **$p < 0.01$, and ***$p < 0.001$).

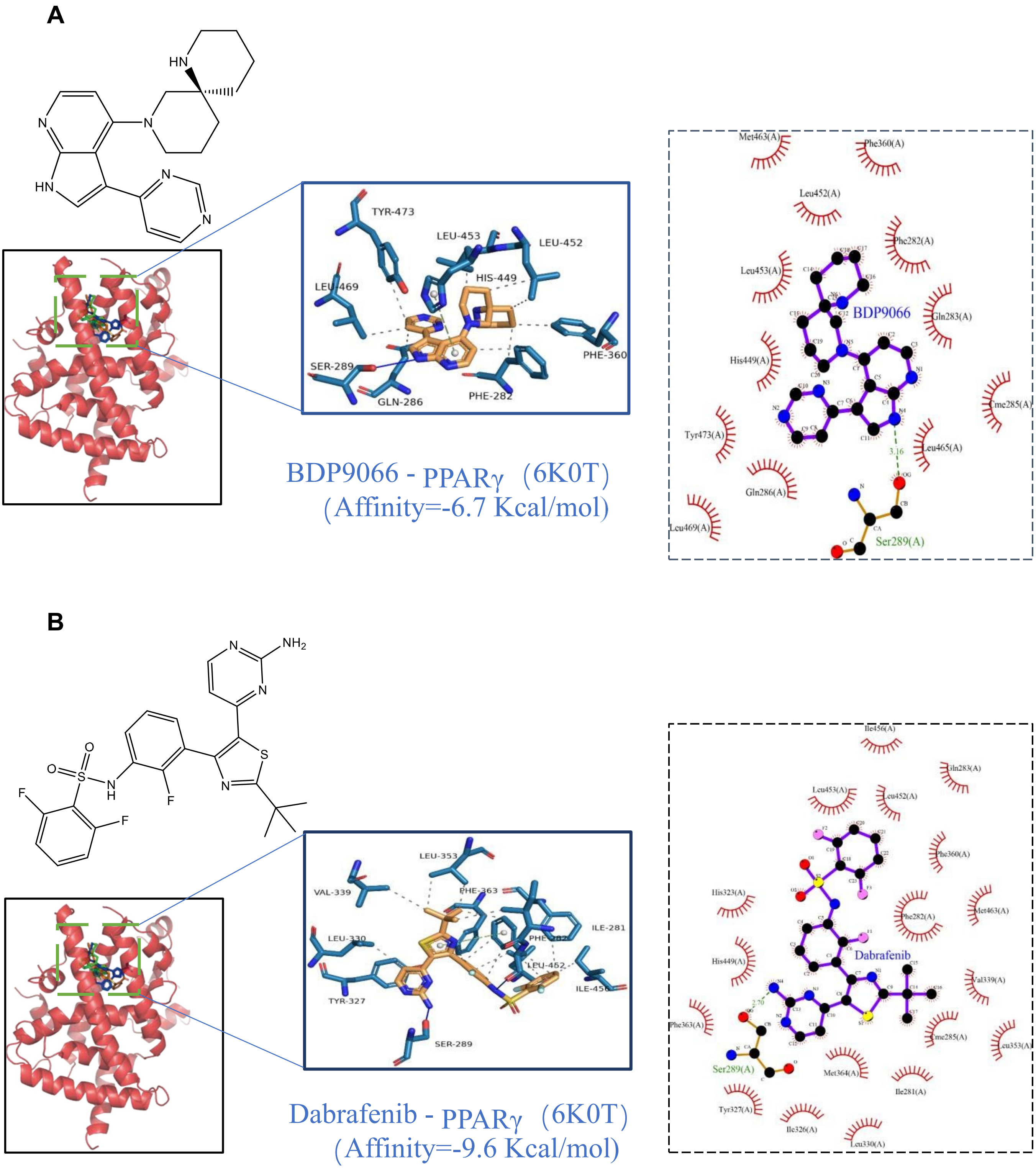

Supplementary Figure 9. Binding scores and binding conformations of potential therapeutic drugs with PPARγ (PDB: 6K0T) simulated using Autodock Vina. (A) Analysis of the two-dimensional binding mode of Nilotinib with 6K0T using LigPlot+ software (left panel) and the three-dimensional binding conformation using Pymol (right panel), with a binding score of -6.7 kcal/mol. (B) Analysis of the two-dimensional binding mode of Axitinib with 6K0T using LigPlot+ software (left panel) and analysis of their three-dimensional binding conformation using Pymol (right panel), with a binding score of -9.6 kcal/mol.

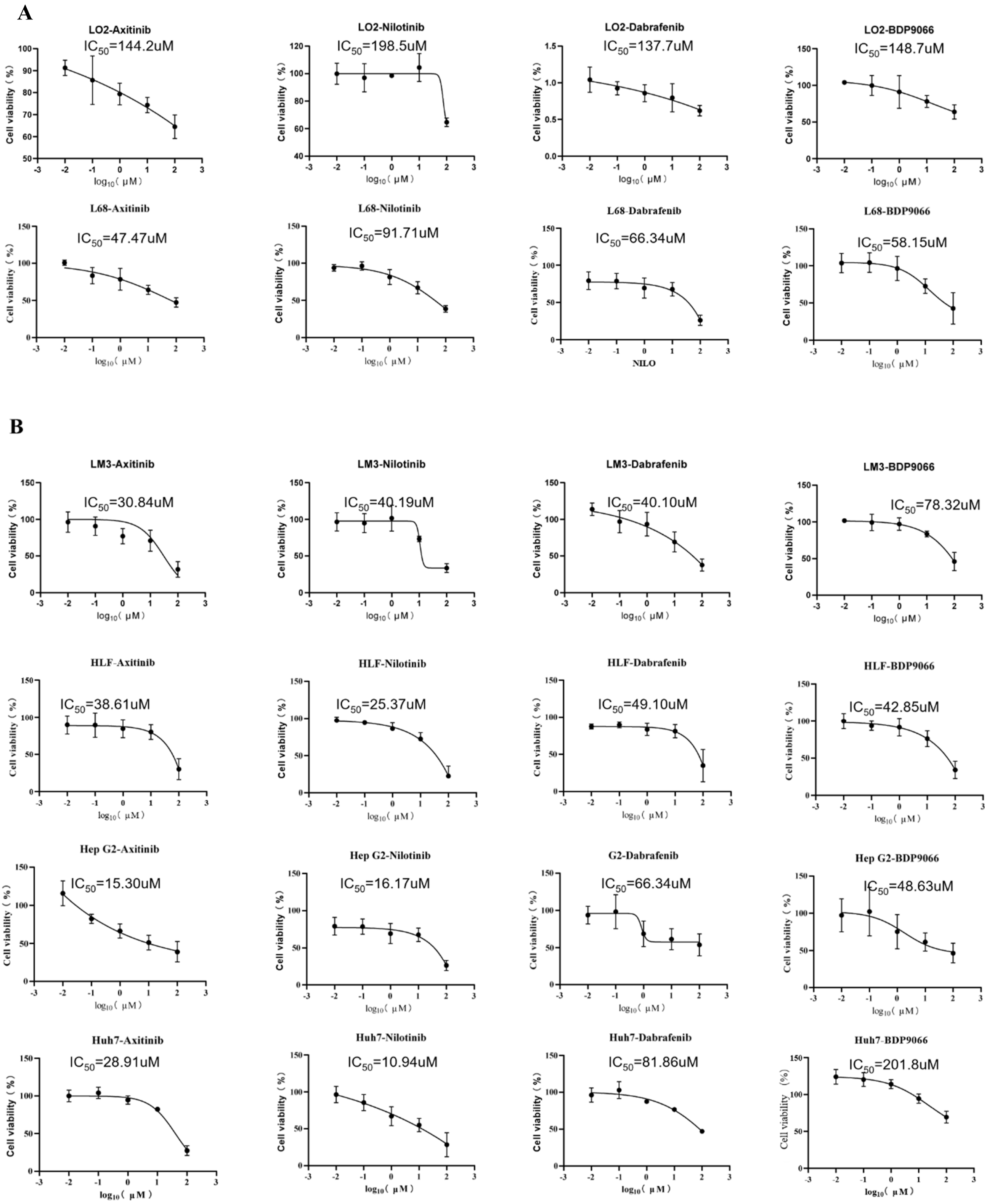

Supplementary Figure 10. (A-B) Fitting of Half Maximal Inhibitory Concentration (IC_{50}) of four drugs in control liver cells and liver cancer cells using GraphPad software.

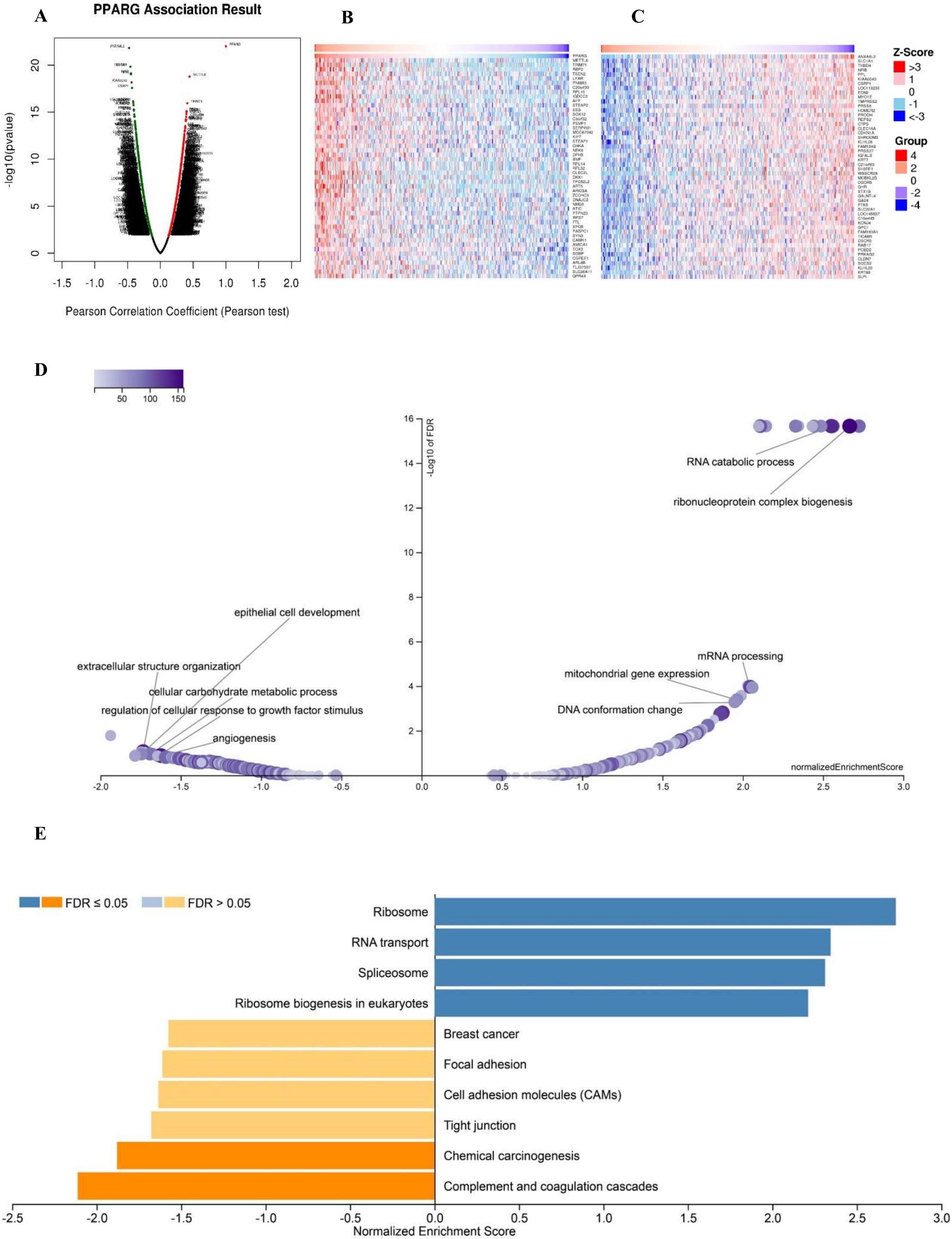

Supplementary Figure 11. PPARG co-expression genes in PAAD analyzed by the LinkedOmics database. (A) Highly relevant genes for PPARG expression in the PAAD cohort examined by Pearson's test. Top 50 positive co-expression genes (B) and negative co-expression

genes (C) of in heat map in PAAD. (D) Volcano plot of PPARG KEGG pathways in PAAD cohort. (E) Directed acyclic graph of PPARG GO analysis (biological process) in PAAD cohort.

Supplementary Table 1. Top 50 genes positively (A) and negatively (B) correlated with PPARG in LIHC.

A

Query	Statistic	P-value	FDR (BH)
PPARG	1	1.00E-43	1.00E-39
MST1R	0.814382	1.88E-43	1.85E-39
ZDHHC3	0.803759	1.52E-41	1.00E-37
KCNN4	0.792476	1.21E-39	6.00E-36
VILL	0.784874	2.00E-38	7.90E-35
PLEK2	0.783916	2.82E-38	9.30E-35
GPRC5A	0.774224	8.40E-37	2.37E-33
S100P	0.76879	5.24E-36	1.29E-32
LGALS3	0.763511	2.96E-35	6.49E-32
VASP	0.762394	4.24E-35	8.38E-32
AIM1L	0.760596	7.54E-35	1.36E-31
TMPRSS4	0.758048	1.69E-34	2.79E-31
KLF5	0.75756	1.97E-34	3.00E-31
RHBDL2	0.757079	2.29E-34	3.24E-31
SERPINB5	0.756438	2.80E-34	3.69E-31
S100A11	0.755012	4.37E-34	5.40E-31
MALL	0.751889	1.14E-33	1.33E-30
MYD88	0.747363	4.49E-33	4.94E-30
EPHA2	0.747155	4.78E-33	4.98E-30
VSIG2	0.745802	7.16E-33	7.08E-30
ACY1	0.745481	7.87E-33	7.12E-30
S100A10	0.745395	8.07E-33	7.12E-30
PTK6	0.745311	8.28E-33	7.12E-30
LAMB3	0.7443	1.12E-32	8.91E-30
ASAP2	0.74427	1.13E-32	8.91E-30
SFN	0.743995	1.22E-32	9.29E-30
OSBPL3	0.742843	1.71E-32	1.26E-29
GJB3	0.742409	1.95E-32	1.37E-29
TMC7	0.742225	2.05E-32	1.40E-29
C19orf33	0.740743	3.16E-32	2.08E-29
FER1L4	0.737413	8.25E-32	5.26E-29
EPS8L1	0.734817	1.72E-31	1.07E-28
TMEM92	0.73154	4.32E-31	2.59E-28
PVRL4	0.730156	6.35E-31	3.69E-28
ITGB4	0.728436	1.02E-30	5.76E-28
S100A16	0.727414	1.35E-30	7.41E-28
PLCD3	0.726306	1.82E-30	9.75E-28
C1orf106	0.724771	2.76E-30	1.44E-27
ANXA2	0.724423	3.04E-30	1.54E-27
HK2	0.723786	3.61E-30	1.78E-27
KRT19	0.723575	3.82E-30	1.84E-27
TINAGL1	0.723145	4.28E-30	2.02E-27
ADAMTSL5	0.722749	4.76E-30	2.19E-27
TRIP10	0.722249	5.44E-30	2.45E-27
RNF149	0.721692	6.31E-30	2.77E-27
UGT1A10	0.720277	9.20E-30	3.95E-27
ARHGAP27	0.718277	1.56E-29	6.47E-27
CAPG	0.718247	1.57E-29	6.47E-27
SH2D3A	0.71751	1.90E-29	7.69E-27
INPP4B	0.715678	3.07E-29	1.21E-26

B

Query	Statistic	P-value	FDR (BH)
PPARG	1	1.00E-43	1.00E-39
MST1R	0.814382	1.88E-43	1.85E-39
ZDHHC3	0.803759	1.52E-41	1.00E-37
KCNN4	0.792476	1.21E-39	6.00E-36
VILL	0.784874	2.00E-38	7.90E-35
PLEK2	0.783916	2.82E-38	9.30E-35
GPRC5A	0.774224	8.40E-37	2.37E-33
S100P	0.76879	5.24E-36	1.29E-32
LGALS3	0.763511	2.96E-35	6.49E-32
VASP	0.762394	4.24E-35	8.38E-32
AIM1L	0.760596	7.54E-35	1.36E-31
TMPRSS4	0.758048	1.69E-34	2.79E-31
KLF5	0.75756	1.97E-34	3.00E-31
RHBDL2	0.757079	2.29E-34	3.24E-31
SERPINB5	0.756438	2.80E-34	3.69E-31
S100A11	0.755012	4.37E-34	5.40E-31
MALL	0.751889	1.14E-33	1.33E-30
MYD88	0.747363	4.49E-33	4.94E-30
EPHA2	0.747155	4.78E-33	4.98E-30
VSIG2	0.745802	7.16E-33	7.08E-30
ACY1	0.745481	7.87E-33	7.12E-30
S100A10	0.745395	8.07E-33	7.12E-30
PTK6	0.745311	8.28E-33	7.12E-30
LAMB3	0.7443	1.12E-32	8.91E-30
ASAP2	0.74427	1.13E-32	8.91E-30
SFN	0.743995	1.22E-32	9.29E-30
OSBPL3	0.742843	1.71E-32	1.26E-29
GJB3	0.742409	1.95E-32	1.37E-29
TMC7	0.742225	2.05E-32	1.40E-29
C19orf33	0.740743	3.16E-32	2.08E-29
FER1L4	0.737413	8.25E-32	5.26E-29
EPS8L1	0.734817	1.72E-31	1.07E-28
TMEM92	0.73154	4.32E-31	2.59E-28
PVRL4	0.730156	6.35E-31	3.69E-28
ITGB4	0.728436	1.02E-30	5.76E-28
S100A16	0.727414	1.35E-30	7.41E-28
PLCD3	0.726306	1.82E-30	9.75E-28
C1orf106	0.724771	2.76E-30	1.44E-27
ANXA2	0.724423	3.04E-30	1.54E-27
HK2	0.723786	3.61E-30	1.78E-27
KRT19	0.723575	3.82E-30	1.84E-27
TINAGL1	0.723145	4.28E-30	2.02E-27
ADAMTSL5	0.722749	4.76E-30	2.19E-27
TRIP10	0.722249	5.44E-30	2.45E-27
RNF149	0.721692	6.31E-30	2.77E-27
UGT1A10	0.720277	9.20E-30	3.95E-27
ARHGAP27	0.718277	1.56E-29	6.47E-27
CAPG	0.718247	1.57E-29	6.47E-27
SH2D3A	0.71751	1.90E-29	7.69E-27
INPP4B	0.715678	3.07E-29	1.21E-26

Supplementary Table 2. The top 50 genes positively (A) and negatively (B) correlated with PPARG in PAAD.

A

Query	Statistic	P-value	FDR (BH)
PPARG	1	1.00E-22	1.00E-18
METTL6	0.445663	1.67E-19	4.76E-16
TRIM71	0.412433	1.14E-16	1.62E-13
RBP2	0.401661	8.14E-16	8.03E-13
TSEN2	0.401443	8.47E-16	8.03E-13
LYAR	0.400709	9.65E-16	8.74E-13
PNMA3	0.397849	1.60E-15	1.39E-12
C20orf30	0.393939	3.19E-15	2.35E-12
RPL15	0.390318	5.98E-15	4.25E-12
IGDCC3	0.389726	6.62E-15	4.55E-12
AFP	0.389381	7.02E-15	4.66E-12
STEAP2	0.388197	8.61E-15	5.47E-12
SSB	0.380567	3.12E-14	1.50E-11
SOX12	0.378601	4.33E-14	1.93E-11
C3orf32	0.378534	4.38E-14	1.93E-11
PSMF1	0.378422	4.46E-14	1.93E-11
SERPINI1	0.377272	5.39E-14	2.24E-11
MGC87042	0.374036	9.17E-14	3.58E-11
KIF7	0.373052	1.08E-13	4.09E-11
STEAP1	0.372978	1.09E-13	4.09E-11
CHKA	0.369094	2.04E-13	7.25E-11
NEK6	0.367811	2.50E-13	8.31E-11
DPH3	0.367545	2.61E-13	8.53E-11
BMF	0.366192	3.24E-13	1.02E-10
RPL14	0.364985	3.92E-13	1.21E-10
RPL32	0.36485	4.00E-13	1.21E-10
CLEC2L	0.364362	4.33E-13	1.29E-10
DKK1	0.362443	5.85E-13	1.69E-10
TPD52L2	0.361596	6.68E-13	1.90E-10
ART5	0.359935	8.65E-13	2.36E-10
ARID3A	0.358857	1.02E-12	2.65E-10
ZCCHC3	0.358838	1.02E-12	2.65E-10
DNAJC2	0.356049	1.57E-12	3.87E-10
NME6	0.355373	1.75E-12	4.24E-10
ATIC	0.355137	1.81E-12	4.34E-10
PTPN23	0.354408	2.02E-12	4.69E-10
RPS7	0.353488	2.33E-12	5.27E-10
FTL	0.352571	2.67E-12	5.98E-10
XPO5	0.351331	3.22E-12	7.12E-10
PABPC1	0.350757	3.51E-12	7.53E-10
SYN3	0.349761	4.08E-12	8.56E-10
CAMK1	0.347722	5.53E-12	1.11E-09
AMICA1	0.346977	6.18E-12	1.20E-09
TOX3	0.346891	6.26E-12	1.20E-09
SOBP	0.346887	6.26E-12	1.20E-09
CGREF1	0.346834	6.31E-12	1.20E-09
ARL8B	0.346811	6.33E-12	1.20E-09
FLJ37307	0.346673	6.46E-12	1.21E-09
SLC26A11	0.346026	7.11E-12	1.30E-09
GPR44	0.345366	7.84E-12	1.42E-09

B

Query	Statistic	P-value	FDR (BH)
PPARG	1	1.00E-22	1.00E-18
METTL6	0.445663	1.67E-19	4.76E-16
TRIM71	0.412433	1.14E-16	1.62E-13
RBP2	0.401661	8.14E-16	8.03E-13
TSEN2	0.401443	8.47E-16	8.03E-13
LYAR	0.400709	9.65E-16	8.74E-13
PNMA3	0.397849	1.60E-15	1.39E-12
C20orf30	0.393939	3.19E-15	2.35E-12
RPL15	0.390318	5.98E-15	4.25E-12
IGDCC3	0.389726	6.62E-15	4.55E-12
AFP	0.389381	7.02E-15	4.66E-12
STEAP2	0.388197	8.61E-15	5.47E-12
SSB	0.380567	3.12E-14	1.50E-11
SOX12	0.378601	4.33E-14	1.93E-11
C3orf32	0.378534	4.38E-14	1.93E-11
PSMF1	0.378422	4.46E-14	1.93E-11
SERPINI1	0.377272	5.39E-14	2.24E-11
MGC87042	0.374036	9.17E-14	3.58E-11
KIF7	0.373052	1.08E-13	4.09E-11
STEAP1	0.372978	1.09E-13	4.09E-11
CHKA	0.369094	2.04E-13	7.25E-11
NEK6	0.367811	2.50E-13	8.31E-11
DPH3	0.367545	2.61E-13	8.53E-11
BMF	0.366192	3.24E-13	1.02E-10
RPL14	0.364985	3.92E-13	1.21E-10
RPL32	0.36485	4.00E-13	1.21E-10
CLEC2L	0.364362	4.33E-13	1.29E-10
DKK1	0.362443	5.85E-13	1.69E-10
TPD52L2	0.361596	6.68E-13	1.90E-10
ART5	0.359935	8.65E-13	2.36E-10
ARID3A	0.358857	1.02E-12	2.65E-10
ZCCHC3	0.358838	1.02E-12	2.65E-10
DNAJC2	0.356049	1.57E-12	3.87E-10
NME6	0.355373	1.75E-12	4.24E-10
ATIC	0.355137	1.81E-12	4.34E-10
PTPN23	0.354408	2.02E-12	4.69E-10
RPS7	0.353488	2.33E-12	5.27E-10
FTL	0.352571	2.67E-12	5.98E-10
XPO5	0.351331	3.22E-12	7.12E-10
PABPC1	0.350757	3.51E-12	7.53E-10
SYN3	0.349761	4.08E-12	8.56E-10
CAMK1	0.347722	5.53E-12	1.11E-09
AMICA1	0.346977	6.18E-12	1.20E-09
TOX3	0.346891	6.26E-12	1.20E-09
SOBP	0.346887	6.26E-12	1.20E-09
CGREF1	0.346834	6.31E-12	1.20E-09
ARL8B	0.346811	6.33E-12	1.20E-09
FLJ37307	0.346673	6.46E-12	1.21E-09
SLC26A11	0.346026	7.11E-12	1.30E-09
GPR44	0.345366	7.84E-12	1.42E-09
